# Automated extraction of leaf mass per area from digitized herbarium specimens

**DOI:** 10.1111/nph.70292

**Published:** 2025-06-18

**Authors:** Thais Vasconcelos, William N. Weaver, Aly Baumgartner, Zoë Bugnaski, James Boyko

**Affiliations:** ^1^ Department of Ecology and Evolutionary Biology University of Michigan Ann Arbor MI 48109 USA; ^2^ University of Michigan Herbarium, University of Michigan Ann Arbor MI 48108 USA; ^3^ Michigan Institute of Data Science University of Michigan Ann Arbor MI 48109 USA

**Keywords:** angiosperms, leaf area, leaf economic spectrum, leaf mass, machine learning, precipitation, solar radiation

## Abstract

- The digitization of vast herbarium collections has made millions of plant specimen images freely available online, which can now be used to generate phenotypic datasets of unprecedented scope. Here, we assess the potential of computer vision tools to automate the extraction of predicted leaf mass per area (LMA_pred_) from digitized herbarium specimens.
- We use an automated pipeline to extract leaf area and petiole width from 22 680 leaves, representing a phylogenetic informed sample of 1580 species of woody angiosperms. LMA_pred_ is estimated using a proxy equation that models the scaling relationship between petiole width and leaf mass. We assess potential sources of error in LMA_pred_ estimates and evaluate whether documented LMA–climate patterns are recovered using this dataset and phylogenetic comparative methods.
- Our LMA_pred_ dataset responds mainly to temperature and solar radiation and presents a positive correlation with latitude. The proxy equation, not the automated pipeline, is responsible for most of the error in LMA_pred_ estimates.
- Our pipeline underscores the power of combining herbarium digitization with new techniques for automated trait scoring. The increased size of datasets generated using this tool allows investigation of potential LMA–climate relationships with a geographically balanced sample while also utilizing comprehensive phylogenetic information.

## Introduction

Leaves are the organs responsible for the bulk of photosynthesis in most plant species, which provide them with the energy used in all metabolic functions and play a key role in transforming carbon from the air into carbon that can enter the energy ladder. Local temperature, water availability, and environmental stress can all impact the efficiency of photosynthesis; thus, specific leaf morphologies are expected to be favored through evolution under different climatic conditions (e.g. Peppe *et al*., 2011; Dong *et al*., 2020; Li *et al*., 2020; Baumgartner & Peppe, 2021). Climatic conditions are highly spatially variable and so consequently, are leaf forms. The almost endless variations in leaf morphologies and the tendency to find similar forms in similar habitats have not gone unnoticed by botanists, ecologists, and evolutionary biologists of the past century (e.g. Bailey & Sinnott, 1915, 1916; Axelrod, 1966; Mooney & Dunn, 1970; Parkhurst & Loucks, 1972; Grime, 1977). As a result, there has been substantial interest in understanding how leaf traits correlate with their surrounding physical environment, especially in relation to climate, when analyzing large spatial scales.

This interest has culminated in foundational studies, such as the description of a leaf economic spectrum that is largely generalizable to woody plants (Reich *et al*., 1997; Wright *et al*., 2004). Leaves of different plant species are positioned along the spectrum by how they use key resources, such as water, nutrients, and carbon, the efficiency of their photosynthesis, and the longevity of their leaves. Leaf mass per area (LMA), a measurement that represents how much biomass is contained in a unit of leaf area (Niinemets, 2001), is the leaf trait that most closely correlates with variation along the leaf economic spectrum. Leaves on the ‘slow‐return’ end of the spectrum have a high LMA and tend to be more costly to produce, with higher longevity and greater investment in defense against environmental stressors. This strategy correlates with lower net photosynthetic rate and is more commonly associated with open areas and limited water resources. Conversely, leaves on the ‘fast‐return’ end of the spectrum have a lower LMA and tend to be cheaper to produce, with shorter leaf lifespans. This strategy correlates with higher net photosynthetic rate and is typical of not only species that prioritize rapid growth, such as deciduous species in temperate forests, but also those in habitats where competition for light is intense and fast growth is essential, such as in tropical rainforests (Reich *et al*., 1997, 1998; Wright *et al*., 2002; Poorter *et al*., 2009; Bruelheide *et al*., 2018; Dong *et al*., 2020; Li *et al*., 2020). These general trends in LMA–climate correlations have been consistently observed across taxa and geographical areas, with dozens of studies addressing this topic over the last two decades (e.g. Niinemets, 2001; Wright *et al*., 2005; Shipley *et al*., 2006; Ordoñez *et al*., 2009; Poorter *et al*., 2009; Heberling & Fridley, 2012; Maire *et al*., 2015; Neyret *et al*., 2016; Dong *et al*., 2020; Joswig *et al*., 2022).

Although the relationship between LMA and climate has been thoroughly explored, two main challenges remain in understanding how climate shapes leaf economic traits over space and time. First, while the relationship between leaf traits and environmental variables is consistently recovered, it is often ‘noisy’, with climate having low predictive power over leaf traits (Anderegg, 2023; Butrim *et al*., 2024). This means that while similar leaf forms tend to be found under similar climatic conditions, co‐occurring species may still show considerable variation in their leaf traits, indicating that many leaf strategies can succeed within the same climate. Recent advances in the availability of phylogenetic data for flowering plants (e.g. Smith & Brown, 2018; Zuntini *et al*., 2024) offer new opportunities to test whether accounting for evolutionary relationships improves the predictive power of trait–environment analyses (Ackerly & Reich, 1999). However, fully leveraging recently inferred phylogenies is constrained by the relatively slower expansion of reliable leaf trait datasets. This leads to a second challenge: the existing taxonomic and geographic gap in functional trait knowledge (the ‘Raunkiaer shortfall’; Hortal *et al*., 2015), particularly for taxa occurring in tropical regions (Vasconcelos, 2023). This lack of data may limit our ability to understand globally generalizable processes and patterns in plant ecology and evolution, as our current understanding might be skewed toward observations in temperate areas where much of the data come from (Collen *et al*., 2008; Cornwell *et al*., 2019).

It is in this context that digitized herbarium specimens can have a large impact on advancing research on trait–environment correlations in plants, especially when combined with machine learning tools for automated trait scoring. With the digitization of large herbarium collections, millions of images of plant specimens are now freely available online, representing an unprecedented source of phenotypic data (Heberling, 2022; Davis, 2023). A common challenge in utilizing this resource is the inconsistency in trait measurements and the time required to manually complete these measurements. This process can be prohibitively slow when carried out by hand, but automated techniques could enable the creation of phenotypic datasets on a scale never seen before (Weaver *et al*., 2020; Weaver & Smith, 2023; Boyko, 2024). These new datasets built from digitized specimens can help overcome gaps and reduce taxonomic and geographical biases in previous trait datasets. This has the potential of substantially increasing data available for analyzing functional traits in both quality and quantity – and finally matching trait datasets with the wealth of phylogenetic data now available.

In this study, we assess the potential of herbarium specimen images and recent developments in machine learning tools to assemble large, phylogenetically balanced LMA datasets. LMA, as the name suggests, is usually measured by dividing leaf mass by leaf area, with both measurements taken directly from fresh or dried leaves (Queenborough & Porras, 2014; Heberling, 2022). Although LMA cannot be directly measured from images of herbarium specimens, it can be predicted based on the scaling relationship between petiole width and leaf mass (LMA_pred_, Royer *et al*., 2007). We evaluate the utility of LMA_pred_ datasets to fill geographical gaps in leaf trait knowledge and for analyzing global LMA–climate correlations, while accounting for the common ancestry between the species. Our specific goals with this investigation were the following: (1) to test the power of machine learning tools in automated measurements of relatively small plant structures that can be observed in images of herbarium specimens, that is, petioles, here utilized to estimate a proxy for LMA; (2) to verify whether a herbarium‐based approach of trait scoring can contribute to closing the biodiversity gap in trait data reported for tropical regions; (3) to assess whether previously recognized patterns of LMA–climate relationships are recovered using a LMA_pred_ dataset; and (4) to test whether the weak relationship between climate and LMA in previous studies may have resulted from ignoring phylogenetic relationships in statistical analyses.

## Materials and Methods

### Phylogenetically informed sample

Our focal sample was restricted to woody nonmonocotyledonous angiosperms (more commonly referred to as ‘woody dicots’) for several reasons. First, the method for estimating LMA_pred_ from petiole width for woody dicot angiosperms (Royer *et al*., 2007; see details below) has a large and globally distributed calibration dataset, unlike similar models for herbaceous angiosperms, broadleaf gymnosperms, and ferns (Royer *et al*., 2010; Peppe *et al*., 2014; see table 13.2 in Peppe *et al*., 2018, for a comparison between all these models). In addition, the herbaceous angiosperm model is restricted to nonsessile leaves with a distinct petiole, which excludes graminoids and would need to be determined on a species‐by‐species basis as many herbaceous angiosperms lack a distinct petiole. Finally, the machine learning software used here has not been yet trained to recognize fern or gymnosperm leaves and does not consistently recognize the leaves/petioles of herbaceous angiosperms.

To ensure that species means estimates for LMA_pred_ were accurate and that we could account for within‐species variation in climatic affinities and phenotypes, we also restricted our sample to species that had at least 10 imaged specimens with valid coordinates online. We also selected species that were included in the seed plant phylogeny built with molecular data from Smith & Brown (2018; i.e. GBMB.tre; henceforward the S&B tree), because our chosen analytical framework requires fully bifurcated phylogenetic trees, and those relationships are more trustworthy when molecular data are the basis of the inference.

**Fig. 1:**
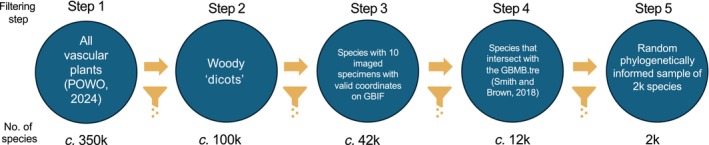
Five steps utilized to choose the sample of species for further measurement and analysis. Step 1: the whole universe of described vascular plants (*c*. 350 k species) in the Plants of the World Online (POWO, 2024) dataset. Step 2: a subset of species in Step 1 that includes only woody ‘dicots’ (*c*. 100 k species). Step 3: a subset of species in Step 2 with at least 10 imaged specimens with valid coordinates available online (*c*. 42 k species). Step 4: a subset of species in Step 3 that were sampled in the Smith & Brown (2018) tree inferred from molecular data (*c*. 12 k species). Step 5: a random, but phylogenetically informed, subset of 2000 species from those available in Step 4. GBIF, Global Biodiversity Information Facility.

To filter a list of only woody dicots, we first gathered all vascular plant names from Plants of the World Online (POWO, (2024), which include 351 028 accepted species (Fig. 1, Step 1). Then, we excluded all the nonangiosperms and monocots from the list, reducing it from a list of 453 families to 342 represented families and 256 471 accepted species. Next, we excluded herbaceous species using the life form data also available through POWO and the categorization of Humphreys *et al*. (2019), which simplifies the Raunkiaer categories of POWO allowing us to filter for species classified under ‘woody perennial’ only. That further reduced our list of species to 117 193 names (Fig. 1, Step 2). We then used the function search_specimen_metadata of R package mvh (Vasconcelos & Boyko, 2025) to filter for taxa with at least 10 herbarium specimens with valid coordinates and images available online, which reduced the list of species to 42 424 names (Fig. 1, Step 3). Next, we intersected this list of species with a version of the S&B tree for which the tips had been taxonomically harmonized to match with the Global Biodiversity Information Facility (GBIF) backbone taxonomy using the R package taxize (Chamberlain & Szöcs, 2013), resulting in a list of 12 195 species (Fig. 1, Step 4).

From the list of 12 195 species left in Step 4, we wanted to sample a subset of species that made sure that all areas of the angiosperm phylogeny were relatively well represented and not biased toward the most species‐rich clades. To that end, we used the S&B tree to create an inverted variance–covariance matrix that was then used to calculate normalized phylogenetic sampling probabilities (i.e. phylogenetic weights) for each of the 12 195 remaining species. Phylogenetic weights were computed following Rohlf (2001), where weights are calculated as (I^T^·C^−1^)·**1** where C^−1^ is the inverse of phylogenetic variance–covariance, I is the identity matrix matching the dimensions of C, and **1** is a vector of ones of length equal to the number of taxa. We then randomly sampled 2000 species from the available taxa, using the phylogenetic weights as sampling probabilities, that is higher weights (more evolutionarily unique taxa) are more likely to be sampled (Fig. 1, Step 5). By picking 2000 species in this step, the goal was to have at least 1500 species left in the final dataset after filtering out inaccurate coordinates, images with poor resolution, and leaves with imprecise segmentation and/or measurement in the LeafMachine2 pipeline (described below).

### Assembly of virtual herbarium

We used the R package mvh (Vasconcelos & Boyko, 2025) to assemble a virtual herbarium of specimens from our target list of 2000 species. We used the function search_specimen_metadata to find records for the species in our list, and removed duplicate records based on their geographic coordinates and records out of the natural range of the genus according to POWO's dataset (POWO, 2024). Low resolution images (< 3 megapixels) can compromise the accuracy of measurements, and specimens imaged without rulers cannot be measured with our pipeline, so images with these characteristics were removed from our final sample as well. Then, for species with more than five imaged specimens left in the metadata after these initial steps of filtering, we set a preference score to favor specimens that came from collections observed to be imaged with rulers that result in more accurate measurements (e.g. rulers with at least two‐unit marker types like cm and mm, or cm and 1/8 inch; Weaver & Smith, 2023) and to avoid specimens from collections observed to have low resolution images online or no ruler on images. Finally, we also favored collections made in the spring and summer for latitudes higher than 30° North and South, to avoid collections from deciduous forests with few or no leaves. We then sampled the five highest records in the preference score of the metadata to download with the function download_specimen_images, downloading a total of 6463 specimens from 136 institutions (GBIF.org, 2025; see Supporting Information Table S1 for list).

### Automated measurements of LMA_pred_ from herbarium specimen images

LMA_pred_ can be estimated from images of herbarium specimens using an equation (henceforward ‘proxy equation’) that is often used by paleobotanists based on the scaling relationship between petiole width and leaf mass:

(Eqn 1) $$\log\left(\mathrm{LMA}\right)=a+b\;\log\left(\text{Petiole}\;{\text{Width}}^{2}/\text{Leaf Area}\right)$$

where for woody dicots *a* = 3.07 and *b* = 0.382, for a predictive power of *R* ^2^ = 0.55 (Royer *et al*., 2007; Peppe *et al*., 2018). This model is based on the correlation between leaf mass and petiole width – the heavier the leaf, the thicker the petiole must be to support it.

**Fig. 2:**
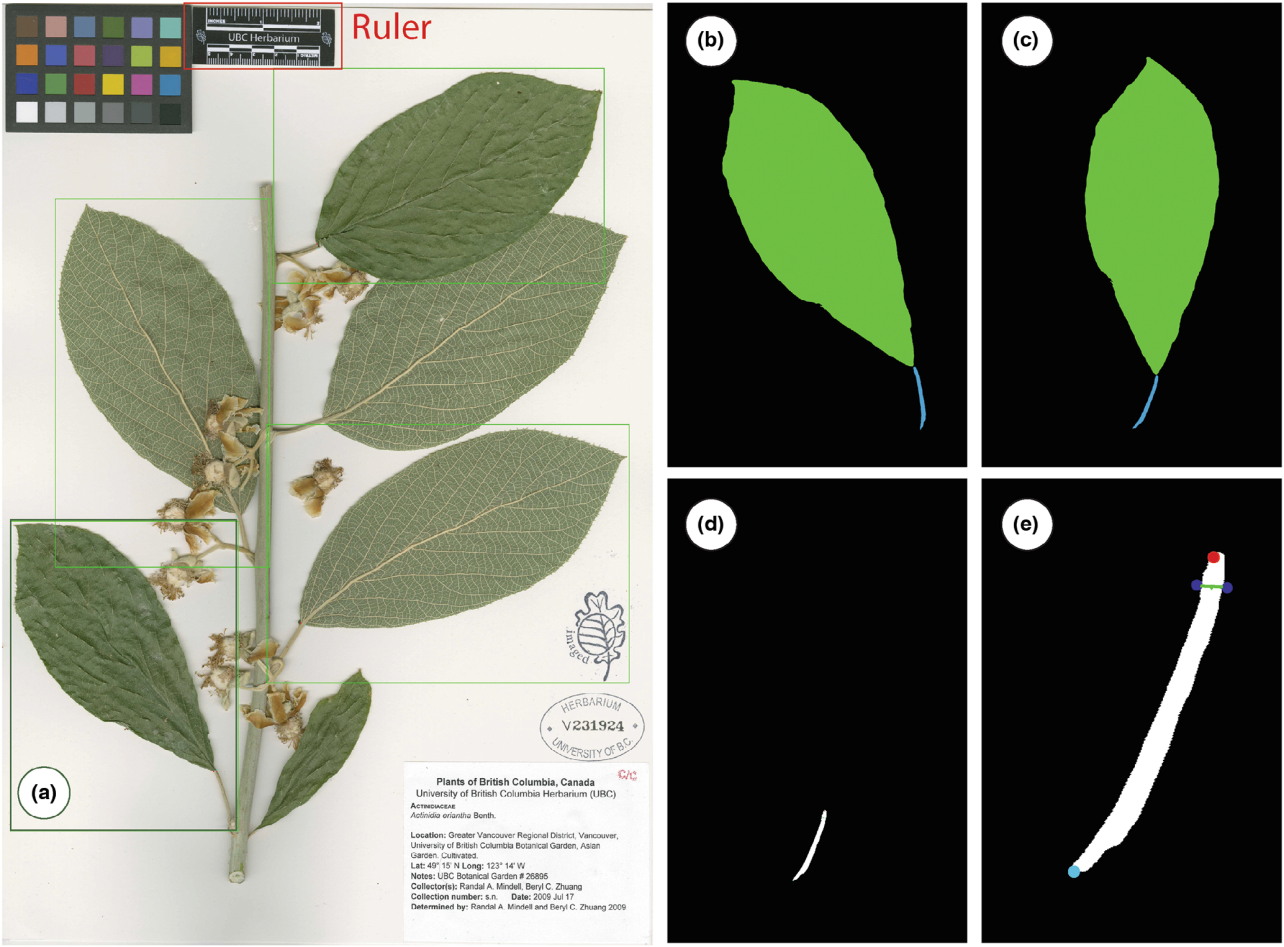
Summarized LeafMachine2 pipeline for measurements of leaf area and petiole width. (a) Identification and masking of leaves and rulers on specimen image. (b) Leaves are segmented, and the area of the leaf (green polygon) is measured based on a pixel to cm conversion factor. (c) Leaves are reoriented based on their shape and likely position of petiole (blue polygon). (d) Petiole is then isolated and (e) measured as the number of pixels in the transversal section of the area close to the base of the leaf (green line). Pixels are then transformed into centimeters based on the same conversion factor as (a).

To automate measurements of leaf area and petiole width, we analyzed the dataset of 6463 specimen images using the instance segmentation model LeafMachine2 v.2.3 (hereafter LM2; Weaver & Smith, 2023), a computer vision tool designed to interpret and process phenotypic data from images of herbarium specimens. For each specimen image, LM2 recognizes, masks, and segments individual leaves and rulers (Fig. 2a). Rulers are used to calculate a conversion factor, that is how many pixels correspond to 1 cm in that image. The area of the leaf is then calculated based on the number of pixels in the segmented leaf mask (Fig. 2b; green polygon). To measure petioles, leaves that were not covered (i.e. bounds of the leaf are unobstructed) or broken were identified. Each of these leaves was then reoriented with the LM2 landmarking model so that the base of each leaf could be consistently located (Fig. 2b,c), and LM2 then identifies the stalk just underneath it as the petiole (Fig. 2c; blue polygon). It then isolates it (Fig. 2d) and calculates the width as the number of pixels on the base of the petiole close to the base of the leaf blade (Fig. 2e), which can then be converted into centimeters (cm) using the conversion factor of the image. It is important to note that the number of leaves per specimen sheet varied widely. For sheets with a single leaf, measurements were straightforward. However, for sheets with multiple leaves, measurements were taken for all identifiable leaves, although not every specimen was of sufficient quality to yield reliable data, and those were discarded at later stages of data processing. Leaflets were treated as equivalent to simple leaves, and petiolules were treated as equivalent to petioles for consistency and as supported by the proxy equation for LMA_pred_ above. To minimize potential biases, leaflets were also excluded from the training dataset of LM2 before analyses to force the software to identify them as simple leaves instead of leaflets.

Because petioles are usually tiny structures that are hard to find and segment, we took additional precautions to ensure that our measurements were reliable. First, as a ground truth for petiole width, we made manual measurements of petioles in 1500 (one to five leaves per sheet) out of the 6463 specimen images in the dataset using Labelbox (Labelbox, 2024) to facilitate cross‐check with automated measurements. A simple linear regression was then performed to assess the strength of the correlation between manual and automated measurements. Second, during initial testing, we discovered that LM2 was suggesting unrealistically large values for petiole width due to specimen shadow in images and certain placement of leaves that compromised measurements (e.g. too close to the edges of the specimen). This led the software to capture the width of the stem instead of the petiole in some instances. After some software adjustment and comparison with ground truth data, we decided to use only the minimum petiole width measurement for each specimen to calculate LMA_pred_ for leaves in that specimen, as this was consistently the closest to the manually measured petioles. We then also removed measurements with a *z*‐score > 3 in the log data per species (all leaves of all specimens in each species) before species‐level analyses.

To quantify how much error comes from the proxy equation and how much comes from LM2 computer vision measurements of petiole widths, we implemented a two‐step Monte Carlo simulation approach. For each leaf in our dataset, we performed 1000 simulations that incorporate uncertainty from: (1) the computer vision measurement error of petiole width and (2) the inherent uncertainty in the proxy equation (Royer *et al*., 2007). First, we quantified measurement uncertainty by applying our calibration model (*R* ^2^ = 0.61; see ‘Summary statistics and computer vision performance’ in the Results section) that converts computer‐vision measured pixel widths to ground truth widths. For each simulation, we added random noise based on the residual SE of this calibration, thereby simulating the measurement error distribution. These simulated widths were then converted from pixels to metric units using the specimen‐specific conversion factors. Second, we propagated these uncertain petiole width measurements through the proxy equation. To account for the inherent uncertainty in this relationship (*R* ^2^ = 0.55), we added normally distributed random errors to each LMA_pred_ value. The error variance was calculated as Var(LMA_pred_) × (1–0.55), representing the unexplained variance in the original regression. This results in a relationship that is expected to produce an *R* ^2^ of 0.55. Finally, for each leaf, we calculated the point estimate of LMA_pred_ alongside 95% confidence intervals derived from the 2.5^th^ and 97.5^th^ percentiles of the simulated distribution.

We then decomposed the relative contributions of different error sources, proxy equation or LM2 petiole measurements, to the total uncertainty in our LMA_pred_ estimates. We conducted three parallel simulations for a random subset of 100 specimens: (1) incorporating uncertainty from both the LM2 petiole measurements and the proxy equation, (2) incorporating uncertainty only from LM2 petiole measurements, and (3) incorporating uncertainty only from the proxy equation. For each scenario, we calculated the variance of the resulting LMA_pred_ distributions and determined the proportion of total variance attributable to each error source.

### Environmental data and LMA_pred_ mapping

Climate data were extracted by overlaying filtered occurrence points from the mvh metadata with high resolution raster files from selected environmental variables. Following previous studies on leaf trait‐climate correlations (e.g. Wright *et al*., 2004, 2017), we extracted climatic data for mean annual temperature (MAT), mean annual precipitation (MAP), temperature seasonality, precipitation seasonality, solar radiation, aridity index, and mean annual wind speed. All variables except the aridity index were taken from 2.5‐min resolution raster layers from WorldClim2 (Fick & Hijmans, 2017), whereas the aridity index was taken from a 30‐s resolution raster (Trabucco & Zomer, 2018). These variables were selected because they represent different axes of climatic variation that may impact leaf morphology, including temperature, water availability (for precipitation variables and aridity index), and physical stress (for mean annual wind speed; Vogel, 2009). Based on preliminary analyses showing a latitudinal gradient in the mapped LMA_pred_, we also included absolute mean latitude as one predictor for multiple regressions analyses, based on the coordinates of specimens sampled for each species in the dataset. Extraction of data per specimen and summarization of climatic means per species were performed in R using functions of the packages geodata, sp, and raster (Hijmans *et al*., 2015, 2024; Pebesma & Bivand, 2005). To visualize the geographical distribution of the sample and distribution of LMA_pred_ in space and per main WWF biome type (Olson *et al*., 2001), we used the R package speciesgeocodeR (Töpel *et al*., 2017) to categorize occurrence points as the biomes where the specimens were originally collected. For analyses of distribution patterns in relation to latitude, we binned mean values of LMA_pred_ and specimen count per 1‐degree latitude before plotting. Finally, to assess the potential of our pipeline to fill geographical and taxonomical gaps in existing open datasets of functional traits, we compare the geographical distribution of the species in our resulting dataset of LMA_pred_ with those currently available on TRY for the trait 3117, ‘Leaf area per leaf dry mass’ (Kattge *et al*., 2020).

### Phylogenetic comparative methods

To evaluate the influence of climatic variables on LMA_pred_, we conducted phylogenetic generalized least squares regressions using the R package phylolm (Ho *et al*., 2016). All analyses were performed on the log‐transformed species‐mean LMA_pred_ values. The phylogeny used for this analysis was a subset of the S&B tree that only included the sampled species. We then used the function dredge from the R package MuMin (Barton & Barton, 2015) to explore all 128 potential combinations of predictors in the full model. Model selection was based on AICc values (top models are presented in Table S3). We standardized climatic variables as *z*‐scores, but little difference was found between standardized and unstandardized measures of climatic variables so only standardized regressions are presented here (see Table S4 for regression results with unstandardized variables). Model averaging was applied to top models within 2 AICc units from the best model to account for model uncertainty. We also used the function extended.pgls of the R package geomorph to analyze the impact of infraspecific variation in univariate regressions between LMA_pred_ and each climatic variable.

To evaluate the influence of phylogenetic relationships on LMA_pred_, we compared the goodness‐of‐fit of predictive models with and without phylogenetic information. Specifically, we examined two measures of *R*‐squared: $R_{\text{resid}}^{2}$ and $R_{\mathrm{lik}}^{2}$ (Ives Anthony, 2019). $R_{\text{resid}}^{2}$ provides an absolute measure of fit and reflects the total variance explained by the model. This includes all fixed effects and the phylogenetic structure. By contrast, $R_{\mathrm{lik}}^{2}$ quantifies the incremental improvement in explanatory power gained by adding phylogenetic structure to the model. We use the rr2 R‐package (Ives & Li, 2018) to examine the different measures of *R* ^2^ in addition to running a complete analysis (including dredge) without phylogenetic information. Finally, we examine the effect sizes and variable importance across the best models. We consider the model‐averaged coefficients as our effect sizes. Variable importance is calculated using the importance function from the MuMin package and is the sum of Akaike weights across all models in which a given predictor appeared.

## Results

### Summary statistics and computer vision performance

**Fig. 3:**
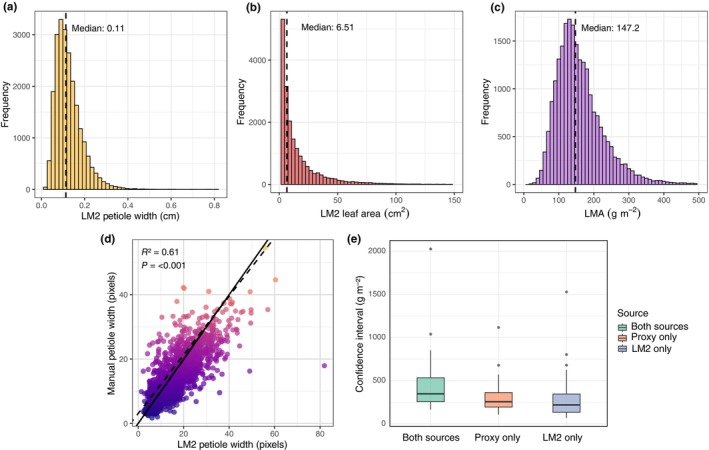
Data distribution for (a) petiole width and (b) leaf area using LM2 in 22 549 leaves where both measurements were recovered (48 outliers omitted in (a) and 2 outliers omitted in (b)). (c) leaf mass per area (LMA) as calculated from LM2 Petiole Width and LM2 Leaf Area using Eqn 1 (42 outliers omitted). Outliers in (a–c) were omitted from figures for visualization purposes only. (d) Comparison between manual and LM2 petiole width measurements in 1755 petioles. Warmer colors represent higher values, and colder color lower values. Solid line in (d) highlights a 1 × 1 relationship. Dashed line indicates the trend line from linear regression in (d) and the median value of the measurements in (a–c). (e) Boxplots comparing the width of 95% confidence intervals when considering different sources of error for predicted leaf mass per area (LMA_pred_).

Our LM2 pipeline recovered measurements for 70 833 leaf areas and 38 079 petiole widths. After all filtering steps that discarded specimens with inaccurate coordinates and leaf areas with no matching petiole and vice versa, we ended up with 22 549 leaves with trustworthy measurements for both leaf area and petiole width (Figs 3a,b, S1, S2), covering a total of 5665 specimens, 1670 species, and 245 angiosperm families (Table S2). To validate our automated approach, we compared a subset of 1755 petiole width measurements that could be accurately measured using both automated and manual methods. This comparison revealed a strong correlation between methods (linear regression, *P* < 0.001, *R* ^2^ = 0.61; Fig. 3d), emphasizing the accuracy of LM2 automated measurements of petiole width. Leaves where both petiole width and leaf area measurements were recovered were used to calculate LMA_pred_, resulting in 22 549 LMA measurements (Figs 3c, S3). Our analysis of sources of error in the estimates showed that measurement uncertainty from LM2 petiole width contributed 41.78% of the total variance, while the proxy equation contributed 58.22% of the variance in the final LMA_pred_ estimates (boxplot comparison, Fig. 3e). The 95% confidence intervals were wider when incorporating both error sources compared with either source alone, but most of the uncertainty came from the proxy relationship (Fig. S4).

### Geographical origin of specimens and spatial distribution of LMA_pred_

**Fig. 4:**
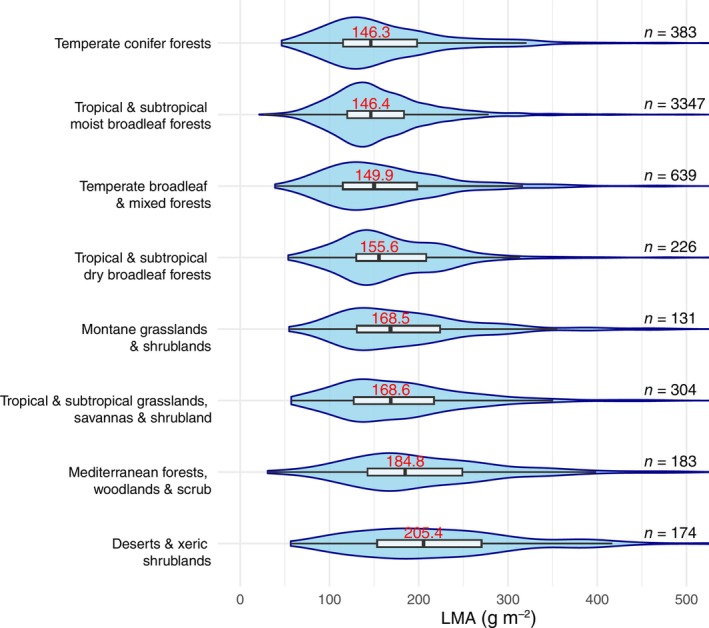
Violin plots showing the density and distribution of sample size (‘*n* = …’ in black, on the right side) and median predicted leaf mass per area (LMA_pred_) values (in red) for biomes with sample size > 100 specimens. Violin plots are ordered from lower to higher LMA_pred_ medians.

**Fig. 5:**
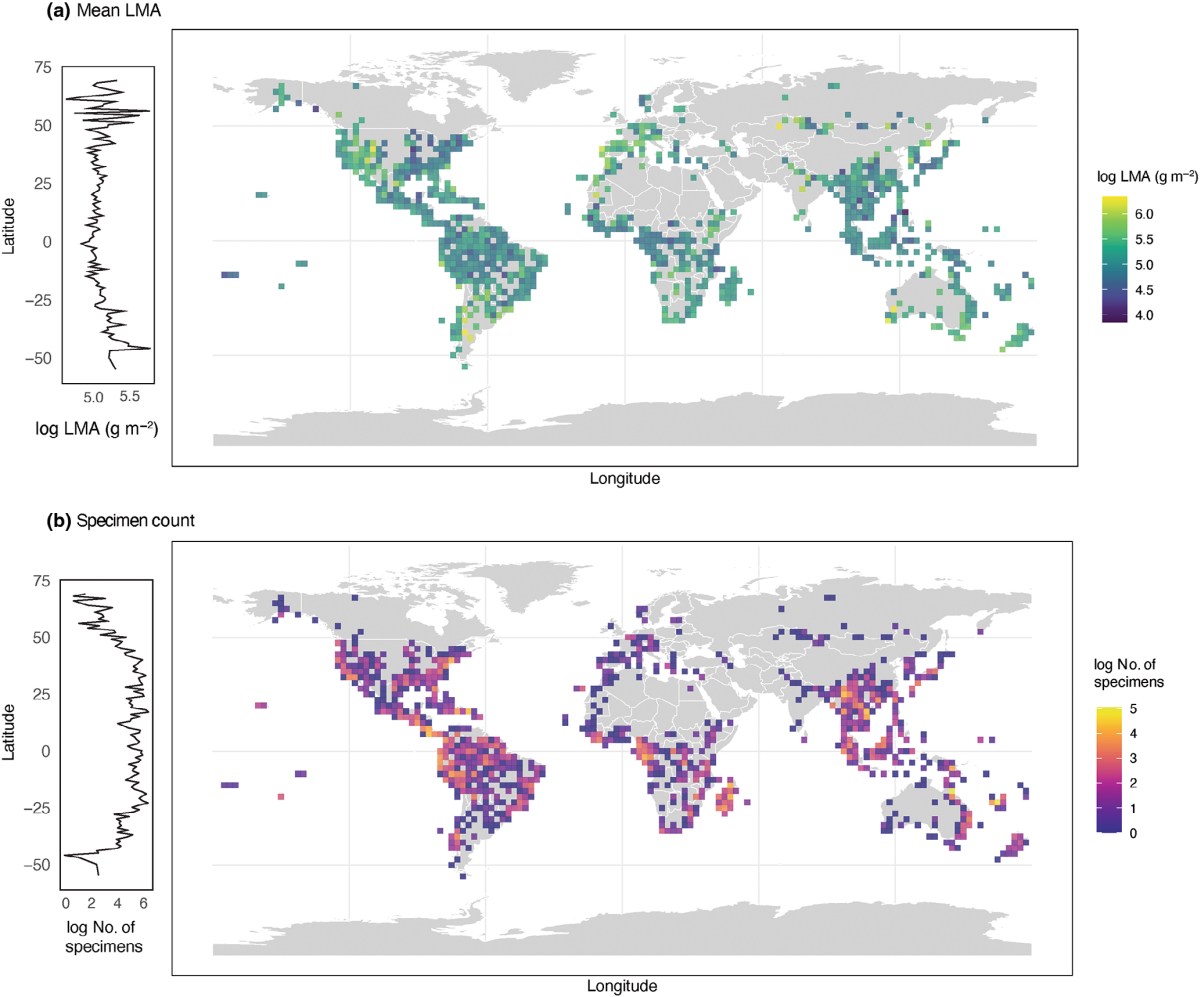
Global distribution of 5665 specimens sampled in this study summarized by (a) mean predicted leaf mass per area (LMA_pred_) and (b) mean specimen count per 2.5 × 2.5 degree grid cell. Both measurements are in log scale.

The best represented biomes in the sample are Tropical & Subtropical Moist Broadleaf Forest (3347 specimens, 59% of the sample), Temperate Broadleaf & Mixed Forest (639, 11%), Temperate Conifer Forest (383, 6.7%), and Tropical & Subtropical Grasslands, Savannas, and Shrublands (304, 5.3%; Fig. 4). The geographical origin of the specimens is relatively well‐distributed across the globe, with particularly good coverage from the tropical Americas, Africa, and Southeast Asia (Fig. 5b). Among the biomes with 100 or more specimens in the sample, LMA_pred_ tends to be highest in Desert & Xeric Shrublands (median 205.4 g m^−2^), Mediterranean Forests, Woodlands & Scrub (184.8 g m^−2^), and Tropical & Subtropical Grasslands, Savannas & Shrublands (168.6 g m^−2^), and lowest in Temperate Conifer Forests (146.3 g m^−2^), Tropical & Subtropical Moist Broadleaf Forest (146.4 g m^−2^), and Temperate Broadleaf & Mixed Forests (149.9 g m^−2^; Figs 4, S5; Notes S1 for all biomes). When analyzing the distribution of LMA_pred_ globally, a positive correlation between LMA_pred_ and latitude is revealed, with lower values of LMA_pred_ tending to be found close to the equator. Larger values are often found in higher latitudes, although the variance is also higher in those regions (Fig. 5a).

### Evolutionary relationships between LMA and climate

**Fig. 6:**
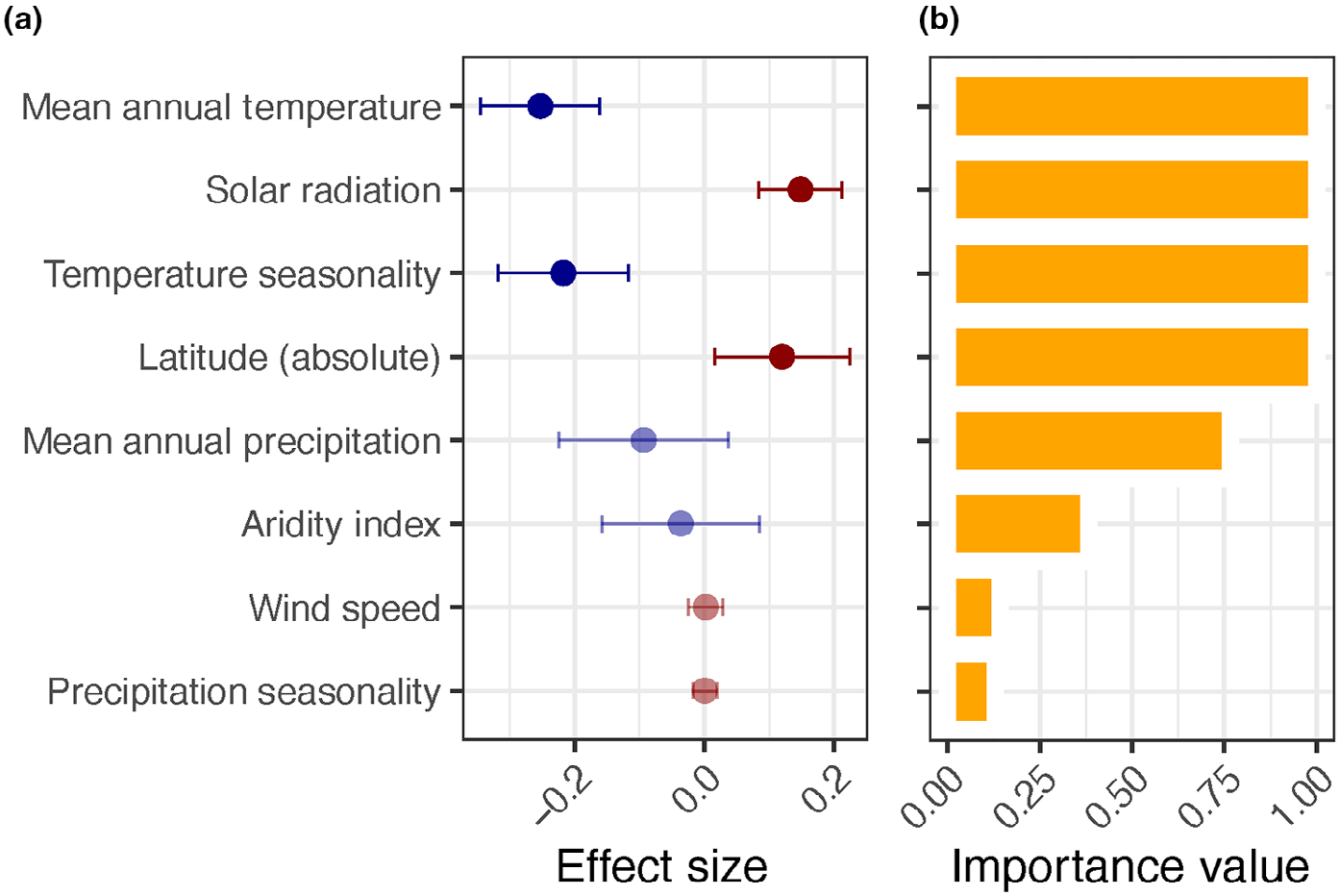
Results of model averaging for the top phylogenetic generalized least squares models. (a) Effect size of each variable in the averaged global phylogenetic model for predicted leaf mass per area (LMA_pred_)–climate correlation, including SE. Red indicates positive effect size (positive correlation), whereas blue indicates negative effect size (negative correlation). Solid dots indicate variables with significant effect on LMA_pred_ (i.e. *P* < 0.001). (b) Importance value of each variable, highlighting how frequently that variable is included as predictors in the models with lowest AICc (higher importance value indicates frequent inclusion of that variable).

The top model (AICc wt = 0.24) of our multiple regression analyses included absolute mean latitude, MAT, MAP, temperature seasonality, and solar radiation as predictors (Tables S3, S4). Our model‐averaged regression found significant effects for MAT (Estimate = −0.253 ± 0.047, *P* < 0.001), solar radiation (0.148 ± 0.032, *P* < 0.001), temperature seasonality (Estimate = −0.217 ± 0.051, *P* < 0.001), and mean absolute latitude (0.119 ± 0.053, *P* < 0.001; Fig. 6a). These three variables were also the ones with the highest importance value (Fig. 6b), that is they are recovered as predictors in all models in the set of best models. MAT and solar radiation presented the largest effect sizes: the first presenting a negative effect and the second two a positive effect on LMA_pred_ (Fig. 6a). Interestingly, when accounting for within‐species variance in univariate analyses, only MAT emerged as significant (Fig. S6). Overall, these results suggest that temperature variables are stronger predictors of LMA_pred_ than precipitation variables at a global scale.

### Model fit and the effect of incorporating phylogenetic information

For the phylogenetic regression model, the $R_{\text{resid}}^{2}$ was 0.293. This means that *c*. 29.3% of the variance in LMA_pred_ was explained by climatic variables when phylogenetic relationships were accounted for. Furthermore, the $R_{\mathrm{lik}}^{2}$ for this model was 0.186. This suggests a significant contribution of phylogeny to the model's explanatory power. By contrast, our re‐analysis using a nonphylogenetic linear model resulted in an $R_{\text{resid}}^{2}$ and $R_{\mathrm{lik}}^{2}$ of 0.122 (there is no difference in the two types of *R* ^2^ because this model assumes no covariance structure in the error). For this model, only 12.2% of the variance was explained. Thus, the inclusion of phylogenetic information resulted in an increase of 42% in explanatory power (from 0.122 to 0.293), suggesting a substantial improvement in model fit when phylogenetic structure is considered. The increase in total variance explained demonstrates the significant role that phylogenetic relationships play in predicting LMA_pred_ alongside climatic variables.

## Discussion

### Machine learning as a tool to unlock herbaria potential as a source of functional trait data

Our approach demonstrates the clear potential of using herbarium specimen images for functional trait scoring. This method can be particularly powerful for addressing taxonomic and geographic gaps in global datasets: even when focusing on a phylogenetically broad but relatively small number of species (< 1% of the total described diversity of angiosperms; POWO, 2024), our sample was surprisingly proportional to species diversity patterns, with most sampled specimens coming from tropical regions and biomes where woody angiosperms are most diverse (Figs 4, 5b; Taylor *et al*., 2023). Also, many of the species we analyzed, even if not the explicit focus of our sampling, are currently absent from the largest and most widely used open dataset of functional traits (i.e. TRY, Kattge *et al*., 2020; Fig. S7). However, it is important to note that, because our LMA_pred_ estimates are derived from digital images of herbarium specimens, rather than from direct measurements of fresh or dried leaves, they differ from conventional LMA measurements usually reported in ecological datasets and should be treated as a distinct trait when exported to open data aggregators of functional traits, and further analyses are needed to understand the implications of integrating these estimates with LMA measurements obtained through other methods. Our work therefore represents an early step toward leveraging automated methods and herbarium specimen images to achieve comprehensive trait diversity knowledge world‐wide. These approaches, combined with the increasing number of imaged collections, have the potential to create datasets far larger than those currently available in online repositories (e.g. BIEN, Maitner *et al*., 2018; TRY, Kattge *et al*., 2020) in the near future.

Our LM2 pipeline had already been demonstrated to work well for leaf area measurements, but for the first time, we attempted to use it to measure petiole width, a structure that is more sensitive to errors due to its small size and similarity to stems. Estimating LMA_pred_ from the automated extraction of petiole width introduces at least two potential sources of error into the estimates: the automated measurement itself, which was shown to have a strong but noisy correlation (*R* ^2^ = 0.61; Fig. 3d) with manual measurements; and the proxy for LMA based on the scaling relationship between petiole width and leaf mass, which again shows a strong but noisy predicting power in the target taxa (*R* ^2^ = 0.55 for woody dicots; Royer *et al*., 2007).

Although our uncertainty analysis revealed that measurement error from the LM2 pipeline contributed to a lower proportion of the total variance than the proxy equation, both had similar contributions to the variance in final LMA_pred_ estimates. In that way, potential paths forward in improving LMA_pred_ estimates using this pipeline should focus both on improving the proxy equation and the automated petiole width measurements. For instance, proxy equations tailored to specific plant lineages within woody dicots could be developed, as the proxy itself likely varies throughout the angiosperm phylogeny. As for the LM2 pipeline, we found that specimens with cordate or irregular bases present greater challenges for accurate petiole width estimation (Table S5; Fig. S8), which could be the focus of future improvements in the computer vision model. Future studies can also use further developments of this method to explore traits like leaf toothiness and shape, and how these contrast with climate.

### Temperature as the main predictor of leaf mass per area

Despite challenges in estimating LMA_pred_ for certain leaf forms, our approach was able to uncover many of the well‐known LMA‐biome and LMA–climate trends previously observed in other studies. For example, we found that lower LMA_pred_ is expected in environments with a high MAT, higher temperature seasonality, and low solar radiation (Fig. 6), which has been shown in previous LMA–climate analyses (e.g. Wright *et al*., 2002; Poorter *et al*., 2009). These conditions are particularly prevalent in tropical and subtropical (low temperature seasonality) and temperate (high temperature seasonality) broadleaf rainforests. As the name suggests, these are environments that favor the colonization and persistence of species with larger leaves. Larger leaves also tend to be relatively thinner and less dense, and to have higher net photosynthetic rates per unit area (Reich *et al*., 1998; Niinemets, 2001; Hassiotou *et al*., 2010). While leaves with these characteristics should be advantageous in areas where competition for light is intense, larger leaves are also thought to be more likely to overheat in hot conditions, especially if not enough water is available in the soil to cool them off through transpiration. Therefore, both water availability and temperature are expected to be important in modulating thermal regulation of a leaf. Without enough water, large leaves risk being damaged by heat (Vogel, 2009; but see Leigh *et al*., 2017).

Therefore, it is surprising that our multiple regression model did not find variables related to precipitation (e.g. MAP and precipitation seasonality) or moisture (e.g. aridity index, wind) as being as explanatory for LMA_pred_ variation as those linked to temperature. While MAP and aridity index appear among the predictors in many of the top models (Table S3), MAT, solar radiation, and temperature seasonality show consistently stronger effects on LMA_pred_ across models (Fig. 6), and only MAT appears to have a significant relationship with LMA_pred_ when within‐species variance is considered (Fig. S6). This result is also unexpected because precipitation is one of the main axes of climatic variation in tropical latitudes (Fick & Hijmans, 2017), which is where most of our sample comes from. Potential explanations for this pattern are that precipitation, and even aridity index, are not ideal proxies for soil water availability, as suggested in previous global analyses (Moles *et al*., 2014). Alternatively, temperature might indeed be the main modulator variable of LMA variability, playing a greater role than water availability in regulating leaf heating and cooling.

### Latitudinal gradient of LMA_pred_

Specimens with lower LMA_pred_ were more frequently found near the equator (Fig. 5b), which aligns with previously documented latitudinal gradients in leaf area (Wright *et al*., 2017), given a general negative correlation is often observed between LMA and leaf area (Wright *et al*., 2004), and the general pattern of low LMA in tropical rainforests (Poorter *et al*., 2009). However, at finer scales, the relationship between leaf area and mass shows variability, as they do not always scale proportionally (e.g. Pickup *et al*., 2005). This variability is consistent with the fact that LMA is influenced by both leaf density and thickness, which can scale differently and independently with climate (Niinemets, 2001). Latitudinal trends in LMA or specific leaf area (SLA, the inverse of LMA) have been observed in local studies (e.g. Wang *et al*., 2016), though sometimes in the opposite direction of our results. Interestingly, our results also show higher LMA_pred_ variance at higher latitudes, which could be due to a smaller sample size from those regions or may reflect true ecological differences. Temperate zones often exhibit variable stress and disturbance patterns, with contrasting strategies like deciduousness (lower LMA) or stress resistance (higher LMA; Poorter *et al*., 2009; see also Fig. S9). Further research in other taxonomic groups not sampled here, such as herbaceous taxa, ferns, and gymnosperms, is needed to determine whether the patterns observed in our study reflect genuine ecological trends or are artifacts of our methodology.

### The role of phylogeny in modulating the leaf investment trade‐off

The increased size of our LMA_pred_ dataset relative to previous work allows us to investigate potential climatic relationships with LMA with a geographically balanced sample and while also utilizing comprehensive phylogenetic information. Leaves are among the most plastic and evolutionarily labile of the plant organs (e.g. Givnish, 2002; Nicotra *et al*., 2011; Chitwood & Sinha, 2016). Indeed, leaf traits are often regarded as poor systematic descriptors at higher taxonomic scales because their morphology and plasticity tend to vary even among closely related lineages (e.g. Royer *et al*., 2008; McKee *et al*., 2019; Baumgartner *et al*., 2020; Gaem *et al*., 2024). This variation results from constant pressure to adapt to changes in the surrounding environment, often in relation to climate, which is the relationship we aimed to explore here. For that reason, it is interesting to see that even at this scale, phylogeny remains important in determining what leaf traits are expected in a given climate. The substantial improvement in model fit with the addition of phylogeny suggests that the regression residuals – errors in the LMA_pred_ based on climatic variables – are not randomly distributed but are instead phylogenetically structured (Ives Anthony, 2019). This means that species within certain parts of the phylogeny tend to have similar deviations (either over‐ or underestimates) from the predicted LMA values. By including phylogeny, the model accounts for these structured residuals, effectively capturing evolutionary patterns that influence LMA beyond the climatic predictors alone.

This structure supports the idea of an evolutionary lag in leaf–climate matching – that is, the tendency of species to inherit traits from ancestors even when these traits may not be the most advantageous in that environment (e.g. Deaner & Nunn, 1999). If evolutionary lag contributes to the limited explanatory power of climate in climate–trait correlations in previous studies (Anderegg, 2023), accounting for phylogenetic relationships in correlation models should strengthen the predictive power of climate in the analyses (e.g. Ackerly & Reich, 1999). When models lack the inclusion of phylogenies, they essentially assume that adaptation occurs instantaneously (Felsenstein, 1985), disregarding the influence of common ancestry on lineage traits and in explaining trait‐environment mismatches. Of course, even when accounting for a phylogenetic structure in the residuals, we should not expect all variation in a trait to be fully explained by the environmental predictors in the regression model. Many additional strategies unrelated to the focal trait likely contribute to a lineage's success in a given environment. In this sense, an ideal LMA value for a particular climate may not exist, as these additional factors also shape a plant's success in an environment.

### Conclusions

Digitizing herbarium specimens is critical for collections to be found and used, and to preserve the data should anything happen to the physical specimen, but developments in machine learning tools are opening them up for new uses. In this study, we demonstrated how machine learning tools enable the assembly of large phylogenetically and geographically balanced datasets by automating trait extraction from images of herbarium specimens. However, despite the potential for machine learning pipelines to enhance trait data gathering, there are several opportunities to refine our approach. Addressing the issue of shadows in smaller leaf structures could greatly enhance measurement accuracy, while the current inability to differentiate leaf developmental stages (i.e. young leaves vs fully expanded leaves) may also introduce a potential source of bias. More comparisons between field, physical herbarium specimens, and computer vision measurements from specimen images for specific taxa and plant structures would refine our approach and allow measuring the contribution of the shrinkage effect in specific traits. Similarly, the proxy equation – our primary source of error – may be improved by developing lineage‐specific versions, so incorporating phylogenetic information could strengthen its predictive power. With continued refinement, these tools have the potential to revolutionize how we extract, analyze, and scale plant trait data globally.

## Competing interests

None declared.

## Author contributions

TV and JB designed the study. WNW led software development. TV, AB, ZB and WNW collected the data. TV, WNW and JB analyzed the data. TV and JB wrote the first draft of the manuscript. All authors discussed and interpreted the results and contributed to the final text.

## Disclaimer

The New Phytologist Foundation remains neutral with regard to jurisdictional claims in maps and in any institutional affiliations.

## Supporting information


**Fig. S1** Ranges of within‐species variation for LMApred (*y*‐axis) for species with at least three specimens sampled in our dataset.
**Fig. S2** Distribution of number of analyzed leaves (*y*‐axis) for each species in the dataset.
**Fig. S3** Plot showing a comparison between the predicted LMA values (*y*‐axis) and the absolute error in petiole width measurements for single leaves (*x*‐axis).
**Fig. S4** Relative contributions of uncertainty due to the proxy equation or LM2 estimates of petiole width for a random sample of 100 leaves from our simulation analysis.
**Fig. S5** Distribution of LMA across all biomes, including those with < 100 specimens within them.
**Fig. S6** Once within‐species variance is considered in univariate regressions between LMApred and climatic factors, only MAT (mean annual temperature) remained a significant relationship (*P* < 0.01).
**Fig. S7** The map displays the geographical distribution of species in our dataset that are not currently available in the TRY database for trait 3117, ‘Leaf area per leaf dry mass’ (Kattge *et al*., 2020).
**Fig. S8** Examples of specimens in our dataset belonging to the genera identified in Table S3.
**Fig. S9** Because it is well known that the relationship between LMA and climate are also variable in terms of whether the species is deciduous or evergreen, we combined data available in TRY (Kattge *et al*., 2020), BIEN (Maitner *et al*., 2018) and other previously published datasets (Wright *et al*., 2004; Peppe *et al*., 2011) to gather data on leaf phenology for our species list, recovering data for 751 taxa, about half of the species.
**Notes S1** Additional interpretation of relationships between LMApred values and biomes.
**Table S1** Institutional codes of the 136 herbaria and number of specimens sampled from each of them.
**Table S2** Full results from LM2, including species name, family, specimen according to GBIF ID number, leaf measurements, conversion factors, and climatic variables.
**Table S3** Top four models based on AICc values and standardized climatic variables resulting from dredge search.
**Table S4** Comparison between full model using unstandardized variables and standardized variables.
**Table S5** List of genera with lowest *R* ^2^ values for the correlation between manual and LMA measurements.

Please note: Wiley is not responsible for the content or functionality of any Supporting Information supplied by the authors. Any queries (other than missing material) should be directed to the *New Phytologist* Central Office.

## Data Availability

All data and code used here are available on https://github.com/tncvasconcelos/leaf_vision and in the Supporting Information. GBIF DOI is available in the reference list.

## References

[nph70292-bib-0001] Ackerly DD , Reich PB . 1999. Convergence and correlations among leaf size and function in seed plants: a comparative test using independent contrasts. American Journal of Botany 86: 1272–1281. 10487815

[nph70292-bib-0002] Anderegg LD . 2023. Why can't we predict traits from the environment? New Phytologist 237: 1998–2004. 36308517 10.1111/nph.18586

[nph70292-bib-0003] Axelrod DI . 1966. Origin of deciduous and evergreen habits in temperate forests. Evolution 20: 1–15. 28564755 10.1111/j.1558-5646.1966.tb03339.x

[nph70292-bib-0004] Bailey IW , Sinnott EW . 1915. Investigations on the phylogeny of the angiosperms 5.Foliar evidence as to the ancestry and early climatic environment of the angiosperms. American Journal of Botany 2: 1–22.

[nph70292-bib-0005] Bailey IW , Sinnott EW . 1916. The climatic distribution of certain types of angiosperm leaves. American Journal of Botany 3: 24–39.

[nph70292-bib-0006] Barton K , Barton MK . 2015. *“Package ‘* mumin *’.” v.1.18: 439* . doi: 10.32614/CRAN.package.MuMIn.

[nph70292-bib-0007] Baumgartner A , Donahoo M , Chitwood DH , Peppe DJ . 2020. The influences of environmental change and development on leaf shape in Vitis. American Journal of Botany 107: 676–688. 32270876 10.1002/ajb2.1460 PMC7217169

[nph70292-bib-0008] Baumgartner A , Peppe DJ . 2021. Paleoenvironmental changes in the Hiwegi Formation (lower Miocene) of Rusinga Island, Lake Victoria, Kenya. Palaeogeography, Palaeoclimatology, Palaeoecology 574: 110458.

[nph70292-bib-0009] Boyko J . 2024. SegColR: deep learning for automated segmentation and color extraction. *bioRxiv* . doi: 10.1101/2024.07.28.605475.

[nph70292-bib-0010] Bruelheide H , Dengler J , Purschke O , Lenoir J , Jiménez‐Alfaro B , Hennekens SM , Botta‐Dukát Z , Chytrý M , Field R , Jansen F *et al*. 2018. Global trait–environment relationships of plant communities. Nature Ecology & Evolution 2: 1906–1917. 30455437 10.1038/s41559-018-0699-8

[nph70292-bib-0011] Butrim MJ , Lowe AJ , Currano ED . 2024. Leaf mass per area: An investigation into the application of the ubiquitous functional trait from a paleobotanical perspective. American Journal of Botany 111: e16419. 39397294 10.1002/ajb2.16419

[nph70292-bib-0013] Chamberlain SA , Szöcs E . 2013. taxize: taxonomic search and retrieval in R. F1000Research 2: 191. 24555091 10.12688/f1000research.2-191.v1 PMC3901538

[nph70292-bib-0014] Chitwood DH , Sinha NR . 2016. Evolutionary and environmental forces sculpting leaf development. Current Biology 26: R297–R306. 27046820 10.1016/j.cub.2016.02.033

[nph70292-bib-0015] Collen B , Ram M , Zamin T , McRae L . 2008. The tropical biodiversity data gap: addressing disparity in global monitoring. Tropical Conservation Science 1: 75–88.

[nph70292-bib-0016] Cornwell WK , Pearse WD , Dalrymple RL , Zanne AE . 2019. What we (don't) know about global plant diversity. Ecography 42: 1819–1831.

[nph70292-bib-0017] Davis CC . 2023. The herbarium of the future. Trends in Ecology & Evolution 38: 412–423. 36549958 10.1016/j.tree.2022.11.015

[nph70292-bib-0018] Deaner RO , Nunn CL . 1999. How quickly do brains catch up with bodies? A comparative method for detecting evolutionary lag. Proceedings of the Royal Society of London, Series B: Biological Sciences 266: 687–694. 10.1098/rspb.1999.0690 PMC1689825 10331289

[nph70292-bib-0019] Dong N , Prentice IC , Wright IJ , Evans BJ , Togashi HF , Caddy‐Retalic S , McInerney FA , Sparrow B , Leitch E , Lowe AJ . 2020. Components of leaf‐trait variation along environmental gradients. New Phytologist 228: 82–94. 32198931 10.1111/nph.16558

[nph70292-bib-0020] Felsenstein J . 1985. Phylogenies and the comparative method. The American Naturalist 125: 1–15. 10.1086/703055 31094602

[nph70292-bib-0021] Fick SE , Hijmans RJ . 2017. WorldClim 2: new 1‐km spatial resolution climate surfaces for global land areas. International Journal of Climatology 37: 4302–4315.

[nph70292-bib-0022] Gaem PH , Andrella GC , Maurin O , Bittrich V , Mazine FF , Lucas E , Estanislau do Amaral MDC . 2024. Integrating datasets from herbarium specimens and images to treat a Neotropical myrtle species complex. Annals of Botany: mcae183. doi: 10.1093/aob/mcae183. PMC12259539 39431944

[nph70292-bib-0023] GBIF.org . 2025. GBIF occurrence download . doi: 10.15468/dl.h2k6pw.

[nph70292-bib-0024] Givnish TJ . 2002. Ecological constraints on the evolution of plasticity in plants. Evolutionary Ecology 16: 213–242.

[nph70292-bib-0025] Grime JP . 1977. Evidence for the existence of three primary strategies in plants and its relevance to ecological and evolutionary theory. The American Naturalist 111: 1169–1194.

[nph70292-bib-0027] Hassiotou F , Renton M , Ludwig M , Evans JR , Veneklaas EJ . 2010. Photosynthesis at an extreme end of the leaf trait spectrum: how does it relate to high leaf dry mass per area and associated structural parameters? Journal of Experimental Botany 61: 3015–3028. 20484320 10.1093/jxb/erq128 PMC2892145

[nph70292-bib-0028] Heberling JM . 2022. Herbaria as big data sources of plant traits. International Journal of Plant Sciences 183: 87–118.

[nph70292-bib-0029] Heberling JM , Fridley JD . 2012. Biogeographic constraints on the world‐wide leaf economics spectrum. Global Ecology and Biogeography 21: 1137–1146.

[nph70292-bib-0030] Hijmans RJ , Barbosa M , Ghosh A , Mandel A . 2024. geodata: download geographic data_. R package v.0.6‐2 . https://CRAN.R‐project.org/package=geodata.

[nph70292-bib-0031] Hijmans RJ , Van Etten J , Cheng J , Mattiuzzi M , Sumner M , Greenberg JA , Lamigueiro OP , Bevan A , Racine EB , Shortridge A *et al*. 2015. *Package ‘* raster’ *. R package 734: 473* . doi: 10.32614/CRAN.package.raster.

[nph70292-bib-0032] Ho LST , Ane C , Lachlan R , Tarpinian K , Feldman R , Yu Q , van der Bijl W , Maspons J , Vos R , Ho MLST . 2016. Package ‘phylolm’ . [WWW document] URL http://cran.r‐project.org/web/packages/phylolm/index.html [accessed 28 February 2025].

[nph70292-bib-0033] Hortal J , de Bello F , Diniz‐Filho JAF , Lewinsohn TM , Lobo JM , Ladle RJ . 2015. Seven shortfalls that beset large‐scale knowledge of biodiversity. Annual Review of Ecology, Evolution, and Systematics 46: 523–549.

[nph70292-bib-0034] Humphreys AM , Govaerts R , Ficinski SZ , Nic Lughadha E , Vorontsova MS . 2019. Global dataset shows geography and life form predict modern plant extinction and rediscovery. Nature Ecology & Evolution 3: 1043–1047. 31182811 10.1038/s41559-019-0906-2

[nph70292-bib-0035] Ives AR , Li D . 2018. rr2: an R package to calculate R2s for regression models. Journal of Open Source Software 3: 1028.

[nph70292-bib-0036] Ives Anthony R . 2019. R2s for correlated data: phylogenetic models, lmms, and glmms. Systematic Biology 68: 234–251. 30239975 10.1093/sysbio/syy060

[nph70292-bib-0037] Joswig JS , Wirth C , Schuman MC , Kattge J , Reu B , Wright IJ , Sippel SD , Rüger N , Richter R , Schaepman ME *et al*. 2022. Climatic and soil factors explain the two‐dimensional spectrum of global plant trait variation. Nature Ecology & Evolution 6: 36–50. 34949824 10.1038/s41559-021-01616-8 PMC8752441

[nph70292-bib-0038] Kattge J , Bönisch G , Díaz S , Lavorel S , Prentice IC , Leadley P , Tautenhahn S , Werner GD , Aakala T , Abedi M *et al*. 2020. TRY plant trait database–enhanced coverage and open access. Global Change Biology 26: 119–188. 31891233 10.1111/gcb.14904

[nph70292-bib-0039] Labelbox . 2024. Labelbox . [WWW document] URL https://labelbox.com [accessed 1 September 2024].

[nph70292-bib-0040] Leigh A , Sevanto S , Close JD , Nicotra AB . 2017. The influence of leaf size and shape on leaf thermal dynamics: does theory hold up under natural conditions? Plant, Cell & Environment 40: 237–248. 10.1111/pce.12857 28026874

[nph70292-bib-0041] Li Y , Zou D , Shrestha N , Xu X , Wang Q , Jia W , Wang Z . 2020. Spatiotemporal variation in leaf size and shape in response to climate. Journal of Plant Ecology 13: 87–96.

[nph70292-bib-0042] Maire V , Wright IJ , Prentice IC , Batjes NH , Bhaskar R , van Bodegom PM , Cornwell WK , Ellsworth D , Niinemets Ü , Ordonez A *et al*. 2015. Global effects of soil and climate on leaf photosynthetic traits and rates. Global Ecology and Biogeography 24: 706–717.

[nph70292-bib-0043] Maitner BS , Boyle B , Casler N , Condit R , Donoghue J , Durán SM , Guaderrama D , Hinchliff CE , Jørgensen PM , Kraft NJ *et al*. 2018. The bien r package: a tool to access the Botanical Information and Ecology Network (BIEN) database. Methods in Ecology and Evolution 9: 373–379.

[nph70292-bib-0044] McKee ML , Royer DL , Poulos HM . 2019. Experimental evidence for species‐dependent responses in leaf shape to temperature: Implications for paleoclimate inference. PLoS ONE 14: e0218884. 31226157 10.1371/journal.pone.0218884 PMC6588257

[nph70292-bib-0046] Moles AT , Perkins SE , Laffan SW , Flores‐Moreno H , Awasthy M , Tindall ML , Sack L , Pitman A , Kattge J , Aarssen LW *et al*. 2014. Which is a better predictor of plant traits: temperature or precipitation? Journal of Vegetation Science 25: 1167–1180.

[nph70292-bib-0047] Mooney HA , Dunn EL . 1970. Convergent evolution of Mediterranean‐climate evergreen sclerophyll shrubs. Evolution 24: 292–303. 28565060 10.1111/j.1558-5646.1970.tb01762.x

[nph70292-bib-0048] Neyret M , Bentley LP , Oliveras I , Marimon BS , Marimon‐Junior BH , Almeida de Oliveira E , Barbosa Passos F , Castro Ccoscco R , Dos Santos J , Matias Reis S *et al*. 2016. Examining variation in the leaf mass per area of dominant species across two contrasting tropical gradients in light of community assembly. Ecology and Evolution 6: 5674–5689. 27547346 10.1002/ece3.2281 PMC4983583

[nph70292-bib-0049] Nicotra AB , Leigh A , Boyce CK , Jones CS , Niklas KJ , Royer DL , Tsukaya H . 2011. The evolution and functional significance of leaf shape in the angiosperms. Functional Plant Biology 38: 535–552. 32480907 10.1071/FP11057

[nph70292-bib-0050] Niinemets Ü . 2001. Global‐scale climatic controls of leaf dry mass per area, density, and thickness in trees and shrubs. Ecology 82: 453–469.

[nph70292-bib-0051] Olson DM , Dinerstein E , Wikramanayake ED , Burgess ND , Powell GVN , Underwood EC , D'Amico JA , Itoua I , Strand HE , Morrison JC *et al*. 2001. Terrestrial ecoregions of the world: a new map of life on Earth. Bioscience 51: 933–938.

[nph70292-bib-0052] Ordoñez JC , Van Bodegom PM , Witte JPM , Wright IJ , Reich PB , Aerts R . 2009. A global study of relationships between leaf traits, climate and soil measures of nutrient fertility. Global Ecology and Biogeography 18: 137–149.

[nph70292-bib-0053] Parkhurst DF , Loucks OL . 1972. Optimal leaf size in relation to environment. The Journal of Ecology 60: 505–537.

[nph70292-bib-0054] Pebesma E , Bivand RS . 2005. S classes and methods for spatial data: the sp package. R News 5: 9–13.

[nph70292-bib-0055] Peppe DJ , Baumgartner A , Flynn A , Blonder B . 2018. Reconstructing paleoclimate and paleoecology using fossil leaves. In: Croft DA , Su DF , Simpson SW , eds. Methods in paleoecology: reconstructing Cenozoic terrestrial environments and ecological communities. Cham, Switzerland: Springer, 289–317.

[nph70292-bib-0056] Peppe DJ , Lemons CR , Royer DL , Wing SL , Wright IJ , Lusk CH , Rhoden CH . 2014. Biomechanical and leaf–climate relationships: a comparison of ferns and seed plants. American Journal of Botany 101: 338–347. 24509795 10.3732/ajb.1300220

[nph70292-bib-0057] Peppe DJ , Royer DL , Cariglino B , Oliver SY , Newman S , Leight E , Enikolopov G , Fernandez‐Burgos M , Herrera F , Adams JM *et al*. 2011. Sensitivity of leaf size and shape to climate: global patterns and paleoclimatic applications. New Phytologist 190: 724–739. 21294735 10.1111/j.1469-8137.2010.03615.x

[nph70292-bib-0058] Pickup M , Westoby M , Basden A . 2005. Dry mass costs of deploying leaf area in relation to leaf size. Functional Ecology 19: 88–97.

[nph70292-bib-0059] Poorter H , Niinemets Ü , Poorter L , Wright IJ , Villar R . 2009. Causes and consequences of variation in leaf mass per area (LMA): a meta‐analysis. New Phytologist 182: 565–588. 19434804 10.1111/j.1469-8137.2009.02830.x

[nph70292-bib-0060] POWO . 2024. Plants of the world online. Kew, UK: Facilitated by the Royal Botanic Gardens. https://powo.science.kew.org/ [accessed 1 October 2024].

[nph70292-bib-0061] Queenborough SA , Porras C . 2014. Expanding the coverage of plant trait databases–a comparison of specific leaf area derived from fresh and dried leaves. Plant Ecology and Diversity 7: 383–388.

[nph70292-bib-0062] Reich PB , Ellsworth DS , Walters MB . 1998. Leaf structure (specific leaf area) modulates photosynthesis–nitrogen relations: evidence from within and across species and functional groups. Functional Ecology 12: 948–958.

[nph70292-bib-0064] Reich PB , Walters MB , Ellsworth DS . 1997. From tropics to tundra: global convergence in plant functioning. Proceedings of the National Academy of Sciences of the United States of America 94: 13730–13734. 9391094 10.1073/pnas.94.25.13730 PMC28374

[nph70292-bib-0065] Rohlf FJ . 2001. Comparative methods for the analysis of continuous variables: geometric interpretations. Evolution 55: 2143–2160. 11794776 10.1111/j.0014-3820.2001.tb00731.x

[nph70292-bib-0066] Royer DL , McElwain JC , Adams JM , Wilf P . 2008. Sensitivity of leaf size and shape to climate within *Acer rubrum* and *Quercus kelloggii* . New Phytologist 179: 808–817. 18507771 10.1111/j.1469-8137.2008.02496.x

[nph70292-bib-0067] Royer DL , Miller IM , Peppe DJ , Hickey LJ . 2010. Leaf economic traits from fossils support a weedy habit for early angiosperms. American Journal of Botany 97: 438–445. 21622407 10.3732/ajb.0900290

[nph70292-bib-0068] Royer DL , Sack L , Wilf P , Cariglino B , Lusk CH , Wright IJ , Westoby M , Jordan GJ , Niinemets Ü , Coley PD *et al*. 2007. Fossil leaf economics quantified: calibration, eocene case study, and implications. Paleobiology 33: 574–589.

[nph70292-bib-0070] Shipley B , Lechowicz MJ , Wright I , Reich PB . 2006. Fundamental trade‐offs generating the worldwide leaf economics spectrum. Ecology 87: 535–541. 16602282 10.1890/05-1051

[nph70292-bib-0071] Smith SA , Brown JW . 2018. Constructing a broadly inclusive seed plant phylogeny. American Journal of Botany 105: 302–314. 29746720 10.1002/ajb2.1019

[nph70292-bib-0073] Taylor A , Weigelt P , Denelle P , Cai L , Kreft H . 2023. The contribution of plant life and growth forms to global gradients of vascular plant diversity. New Phytologist 240: 1548–1560. 37264995 10.1111/nph.19011

[nph70292-bib-0074] Töpel M , Zizka A , Calió MF , Scharn R , Silvestro D , Antonelli A . 2017. SpeciesGeoCoder: fast categorization of species occurrences for analyses of biodiversity, biogeography, ecology, and evolution. Systematic Biology 66: 145–151. 27486181 10.1093/sysbio/syw064 PMC5410971

[nph70292-bib-0075] Trabucco A , Zomer RJ . 2018. Global aridity index and potential evapotranspiration (ET0) climate database v.2. CGIAR Consort Spat Inf 10: m9 . 10.1038/s41597-022-01493-1 PMC9287331 35840601

[nph70292-bib-0076] Vasconcelos T . 2023. A trait‐based approach to determining principles of plant biogeography. American Journal of Botany 110: e16127. 36648370 10.1002/ajb2.16127

[nph70292-bib-0077] Vasconcelos T , Boyko JD . 2025. mvh: an R tool to assemble and organize virtual herbaria from openly available specimen images. Applications in Plant Sciences 13: e11631. 40308897 10.1002/aps3.11631 PMC12038725

[nph70292-bib-0078] Vogel S . 2009. Leaves in the lowest and highest winds: temperature, force and shape. New Phytologist 183: 13–26. 19413689 10.1111/j.1469-8137.2009.02854.x

[nph70292-bib-0079] Wang R , Yu G , He N , Wang Q , Zhao N , Xu Z . 2016. Latitudinal variation of leaf morphological traits from species to communities along a forest transect in eastern China. Journal of Geographical Sciences 26: 15–26.

[nph70292-bib-0080] Weaver WN , Ng J , Laport RG . 2020. LeafMachine: using machine learning to automate leaf trait extraction from digitized herbarium specimens. Applications in Plant Sciences 8: e11367. 32626609 10.1002/aps3.11367 PMC7328653

[nph70292-bib-0081] Weaver WN , Smith SA . 2023. From leaves to labels: building modular machine learning networks for rapid herbarium specimen analysis with LeafMachine2. Applications in Plant Sciences 11: e11548. 37915430 10.1002/aps3.11548 PMC10617304

[nph70292-bib-0082] Wright IJ , Dong N , Maire V , Prentice IC , Westoby M , Díaz S , Gallagher RV , Jacobs BF , Kooyman R , Law EA *et al*. 2017. Global climatic drivers of leaf size. Science 357: 917–921. 28860384 10.1126/science.aal4760

[nph70292-bib-0083] Wright IJ , Reich PB , Cornelissen JH , Falster DS , Groom PK , Hikosaka K , Lee W , Lusk CH , Niinemets Ü , Oleksyn J *et al*. 2005. Modulation of leaf economic traits and trait relationships by climate. Global Ecology and Biogeography 14: 411–421.

[nph70292-bib-0084] Wright IJ , Reich PB , Westoby M , Ackerly DD , Baruch Z , Bongers F , Cavender‐Bares J , Chapin T , Cornelissen JH , Diemer M *et al*. 2004. The worldwide leaf economics spectrum. Nature 428: 821–827. 15103368 10.1038/nature02403

[nph70292-bib-0085] Wright IJ , Westoby M , Reich PB . 2002. Convergence towards higher leaf mass per area in dry and nutrient‐poor habitats has different consequences for leaf life span. Journal of Ecology 90: 534–543.

[nph70292-bib-0086] Zuntini AR , Carruthers T , Maurin O , Bailey PC , Leempoel K , Brewer GE , Epitawalage N , Françoso E , Gallego‐Paramo B , McGinnie C *et al*. 2024. Phylogenomics and the rise of the angiosperms. Nature 629: 1–8. 10.1038/s41586-024-07324-0 PMC11111409 38658746

