# Supplementary material for: Automated extraction of leaf mass per area from digitized herbarium specimens
Source: New Phytol. 2025 Jun 18;251(2):896–908. doi: 10.1111/nph.70292 (PMC13278661; doi:10.1111/nph.70292)
Supplement: Supplementary file 1 — Fig. S1 Ranges of within‐species variation for LMApred (y‐axis) for species with at least three specimens sampled in our dataset. Fig. S2 Distribution of number of analyzed leaves (y‐axis) for each species in the dataset. Fig. S3 Plot showing a comparison between the predicted LMA values (y‐axis) and the absolute error in petiole width measurements for single leaves (x‐axis). Fig. S4 Relative contributions of uncertainty due to the proxy equation or LM2 estimates of petiole width for a random sample of 100 leaves from our simulation analysis. Fig. S5 Distribution of LMA across all biomes, including those with < 100 specimens within them. Fig. S6 Once within‐species variance is considered in univariate regressions between LMApred and climatic factors, only MAT (mean annual temperature) remained a significant relationship (P < 0.01). Fig. S7 The map displays the geographical distribution of species in our dataset that are not currently available in the TRY database for trait 3117, ‘Leaf area per leaf dry mass’ (Kattge et al., 2020). Fig. S8 Examples of specimens in our dataset belonging to the genera identified in Table S3. Fig. S9 Because it is well known that the relationship between LMA and climate are also variable in terms of whether the species is deciduous or evergreen, we combined data available in TRY (Kattge et al., 2020), BIEN (Maitner et al., 2018) and other previously published datasets (Wright et al., 2004; Peppe et al., 2011) to gather data on leaf phenology for our species list, recovering data for 751 taxa, about half of the species. Notes S1 Additional interpretation of relationships between LMApred values and biomes. Table S1 Institutional codes of the 136 herbaria and number of specimens sampled from each of them. Table S2 Full results from LM2, including species name, family, specimen according to GBIF ID number, leaf measurements, conversion factors, and climatic variables. Table S3 Top four models based on AICc values and standardized climatic v [file NPH-251-896-s001.pdf]

New Phytologist **Supporting Information**

Article title: Automated extraction of leaf mass per area from digitized herbarium specimens
Authors: Thais Vasconcelos, William N. Weaver, Aly Baumgartner, Zoe Bugnaski, James Boyko
Article acceptance date: 21 May 2025

The following Supporting Information is available for this article:

Table S1) Institutional codes of the 136 herbaria and number of specimens sampled from each of them. Institutional codes follow GBIF format.

INSTITUTION	NUMBER OF SPECIMENS
**NY**	7108
**MNHN**	3163
**US**	2260
**MEISEBG**	1761
**F**	601
**MA**	457
**FLAS**	430
**ASU**	424
**JBRJ**	382
**B**	369
**BRIT**	360
**CAS**	331
**CM**	315
**KAG**	314
**RSA**	314
**UCR**	283
**USF**	280
**K**	270
**Z**	249
**BBM**	203
**LSU**	193
**USCH**	169
**MIN**	165
**UM**	158
**LI**	154
**NCU**	153
**CHSC**	151

**A**	143
**MOAR**	143
**NCSC**	140
**PRC**	140
**CHR**	134
**GA**	113
**VT**	110
**NHMUK**	107
**MU**	97
**NEON**	97
**MOSCOW STATE UNIVERSITY**	94
**OBI**	92
**CLEMS**	78
**DES**	78
**DBG**	73
**NMNZ**	71
**BRY**	65
**AK**	59
**USAC**	58
**UNA**	53
**AU**	49
**MCA**	47
**QARSHI BOTANICAL GARDEN**	46
**COI**	42
**O**	42
**MICH**	41
**GMUF**	40
**ODU**	39
**MVSC**	38
**CINC**	35
**PUA**	33
**DAV**	30
**HSC**	29
**SBBG**	29
**TRH**	29
**MMNS**	28
**TAAM**	28

**UVG**	26
**LOB**	25
**DEK**	24
**OSU**	24
**SDSU**	24
**IRVC**	23
**CMN**	22
**UNM**	22
**ASC**	21
**CS**	21
**DR**	21
**SFV**	21
**SJSU**	21
**WU**	21
**YPM**	21
**EIU**	19
**ZT**	18
**W**	17
**CHRB**	15
**CJBN**	15
**KU**	15
**WS**	15
**GJO**	13
**MISS**	12
**SMF**	12
**MO**	11
**TENN**	11
**ANHC**	10
**CONC**	9
**ISA**	9
**NHMD**	9
**UCSC**	9
**USU**	9
**SBAC**	8
**TU**	8
**E**	7
**HAZARA UNIVERSITY**	7
**IRKUTSK STATE UNIVERSITY**	7
**PI**	7

**POM**	7
**UCSB**	7
**UNIVERSITE DE MONTREAL BIODIVERSITY CENTRE**	7
**CSUSB**	6
**KYO**	6
**FSC**	5
**GBS RAN - GLAVNY BOTANICHESKY SAD ROSSIJSKOJ AKADEMII NAUK**	5
**TI**	5
**TROM**	5
**BAYLU**	4
**BBS**	4
**COLO**	4
**EKY**	4
**HXC**	4
**MOR**	4
**MUHW**	4
**TAC**	4
**UAM**	4
**UCJ**	4
**USZ**	4
**CAU**	3
**H**	2
**LA**	2
**VPI**	2
**BC**	1
**FTG**	1
**MEL**	1
**NSW**	1
**PH**	1
**SNSNMC**	1
**TTC**	1
**USMS**	1
**WCUH**	1

Figure S1: Ranges of within species variation for LMA_{pred} (y-axis) for species with at least three specimens sampled in our dataset. Species in x-axis are ordered by mean LMA_{pred} estimates.

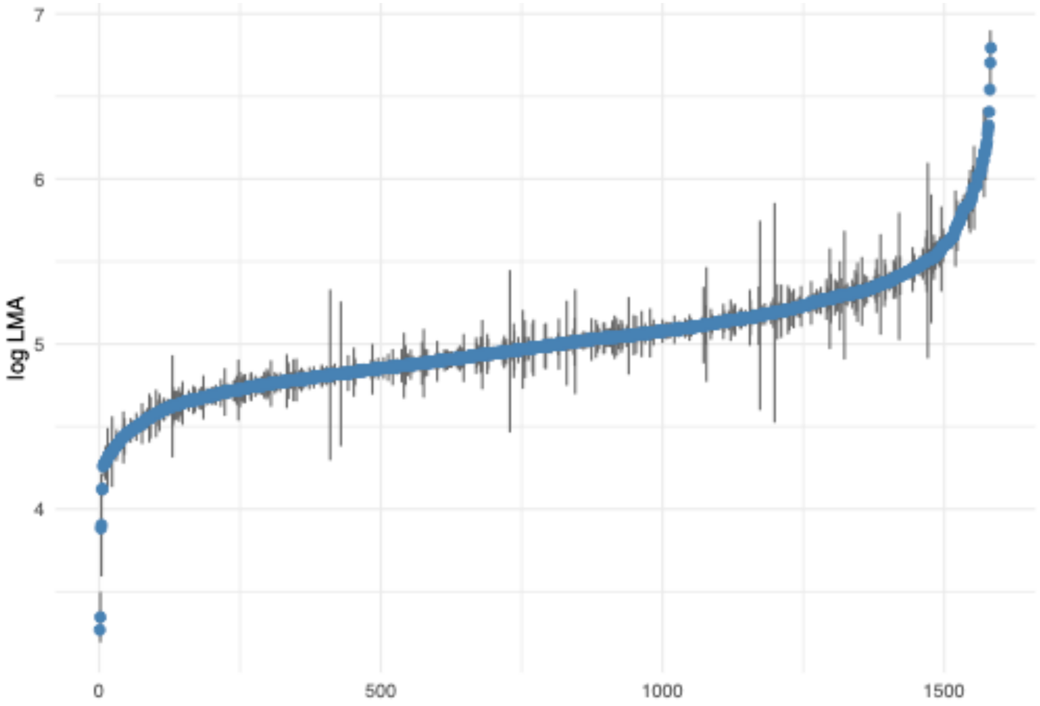

Figure S2: Distribution of number of analyzed leaves (y-axis) for each species in the dataset. Each bar represents the count for a specific species, ordered by the number of observations (x-axis). Black dashed line indicates the median count (median=9).

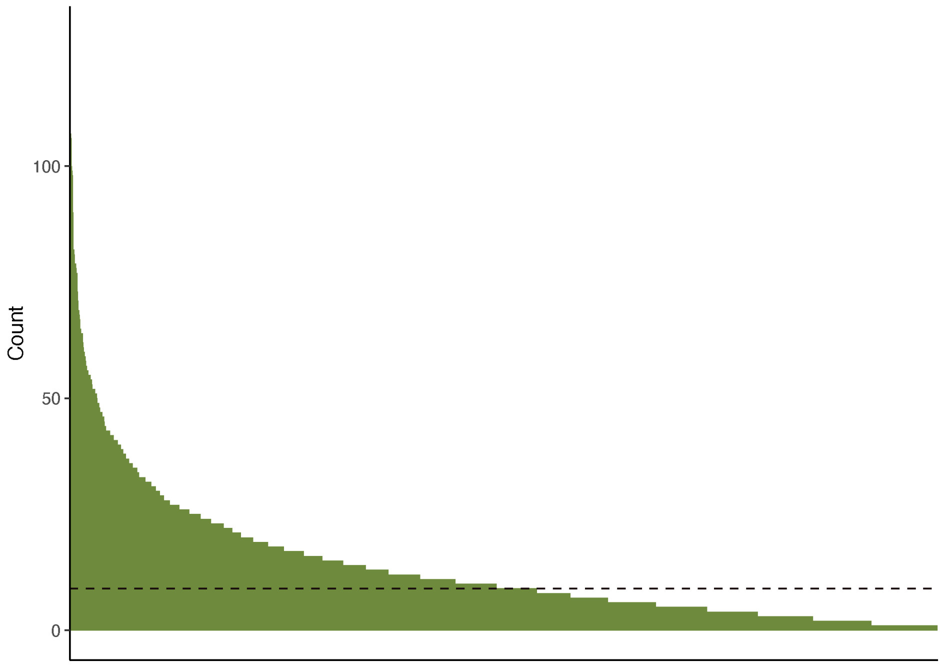

Table S2) (separate .csv file) Full results from LM2 including species name, family, specimen according to GBIF ID number, leaf measurements, conversion factors, and climatic variables. Each row corresponds to a single leaf. To search for specimen on GBIF, use https://www.gbif.org/occurrence/<gbif_id>. Each column in this table contains the following information:

Column	Description
**component_name**	Unique identifier of single leaf in a specimen
**filename**	Unique identifier of specimen, indicating species and gbif Id
**gbif_id**	Unique GBIF identifier
**conversion_mean**	How many pixels correspond to one centimeter (cm) in that image
**predicted_conversion_factor_cm**	How many pixels correspond to one centimeter (cm) in that image – predicted based on the size of specimen and ruler position
**genus_species**	Species analyzed in that row
**family**	Angiosperm family
**area**	Area of that specific leaf based on LM2 measurement and conversion factor
**petiole_width**	Petiole width of that specific leaf based on LM2 measurement and conversion factor
**LMA**	LMA_{pred} estimated based on area, petiole_width and proxy equation of Royer et al. (2007)
**lat**	Latitude where specimen was collected
**lon**	Longitude where specimen was collected
**eco_name**	Eco region where specimen was collected
**biome**	Biome where specimen was collected
**bio_1**	Mean Annual Temperature where specimen was collected
**bio_4**	Temperature Seasonality where specimen was collected
**bio_12**	Mean Annual Precipitation where specimen was collected
**bio_15**	Precipitation Seasonality where specimen was collected
**ai**	Aridity Index where specimen was collected
**wind**	Mean Wind Speed where specimen was collected
**srad**	Solar Radiation where specimen was collected
**leaf_phenology**	Leaf phenology for that species based on TRY dataset (when known)

Figure S3: Plot showing a comparison between the predicted LMA values (y-axis) and the absolute error in petiole width measurements for single leaves (x-axis). It indicates that there is no significant bias in the performance of automated petiole width measurements for specimens with higher or lower LMA values. The color gradient on the plot represents LMA values, with warmer colors corresponding to higher LMA values.

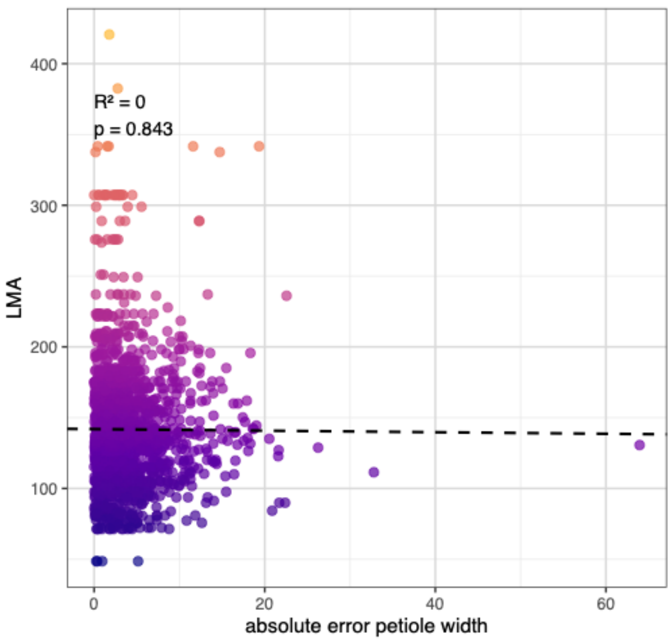

Figure S4. Relative contributions of uncertainty due to the proxy equation or LM2 estimates of petiole width for a random sample of 100 leaves from our simulation analysis. Analysis indicates that the proxy equation contributes the larger proportion of overall uncertainty in LMA estimation but also shows some leaf specific patterns.

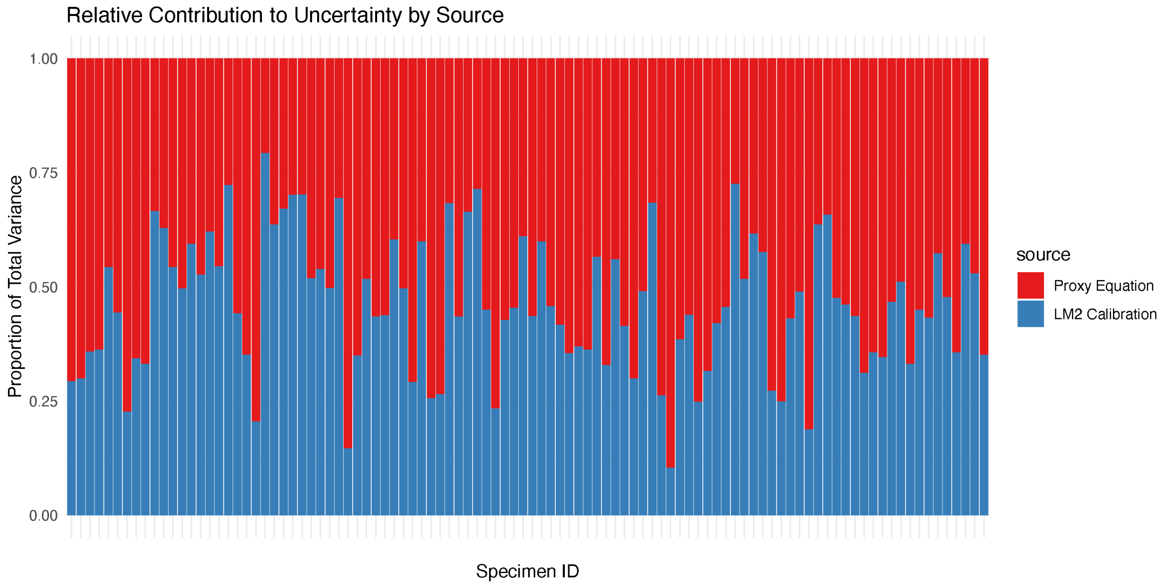

Figure S5) Distribution of LMA across all biomes, including those with less than 100 specimens within them. Red numbers indicate median LMA and black number (right) indicate number of specimens sampled from each biome. Violin plots indicate density of points through the LMA range of values**.**

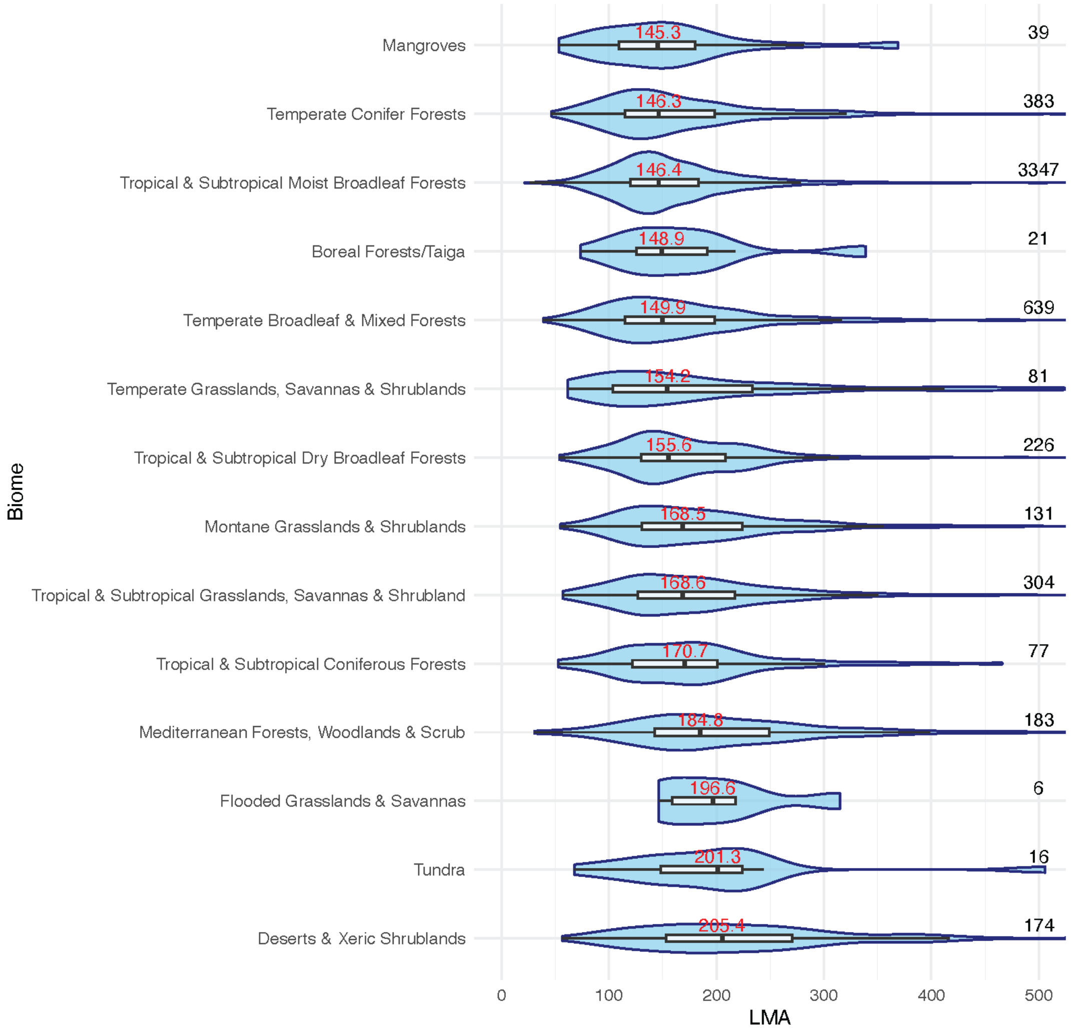

Notes S1) Additional interpretation of relationships between LMA_{pred} values and biomes are as follows: we note that there seem to be instances where our LMA_{pred} estimates are not comparable to those previously reported in similar conditions or in similar groups. Specifically, there is some reason to believe that our estimates of LMA are upwardly biased. For example, Poorter *et al.* (2009), in reviewing the literature of LMA, reports a median of 167 g/m^2 for evergreen shrubs and 106 g/m^2 for evergreen trees; our median for evergreen species in general was in between these two values, but closer to the shrub estimates at 147.2 g/m^2, even though most of our sample comes from forest biomes where supposedly trees species-richness is relatively higher than in non-forest biomes (Taylor et al., 2023). Meanwhile, the same paper reports a median of 73 g/m^2 and 75 g/m^2 for deciduous shrubs and trees, respectively, while our median estimate for deciduous species in general was almost two times as high, at 128.94 g/m^2.

This could be because our automated measurements tend to be more successful for small-leaved trees and shrubs, which may tend to have higher LMA. LM2 tends to work better for leaves that are not covered by any other structure in the specimen image, and this is more likely to be true when the leaf is small. The version of LM2 used here has more larger leaves in its training dataset than the original software, so although this "small-leaf" bias may be less conspicuous than in the original software, it is still possible that many larger leaves were discarded along our pipeline when LM2 could not recognize their leaves due to occlusion. Indeed, our variation in leaf area from 0.01cm^2 in *Arenaria longipedunculata* (Caryophyllaceae) to 717.62 cm^2 in *Piper squamulosum* (Piperaceae), while broad, is still smaller than that reported in nature (Wright *et al*., 2017), which is one of the main limitations of automated methods of leaf trait measurements from herbarium specimens. For larger leaves, future methods could be developed to automate unfolding of leaves or estimate leaf area through vein density (Sack *et al*., 2012), but not with our current pipeline. However, we also note that once leaves are recognized by LM2, than there is no evidence for bias related to leaf size in the accuracy of estimates (Figure S3 above).

Table S3 Top four models based on AICc values and standardized climatic variables resulting from dredge search. Abs_lat = absolute mean latitude, ai = aridity index, bio_1 = Mean Annual Temperature, bio_12 = Mean Annual Precipitation, bio_15 = Precipitation Seasonality, bio_4 = Temperature Seasonality, srad = Solar Radiation, wind = Mean Wind Speed. Top model in the first row is highlighted in blue.

(Intercept)	abs_lat	ai	bio_1	bio_12	bio_15	bio_4	srad	wind	df	logLik	AICc	delta	weight
**-0.155**	**0.19**		**-0.224**	**-0.124**		**-0.257**	**0.152**		**8**	**-2079.965**	**4176.022**	**0**	**0.241**
**-0.15**	0.192	-0.119	-0.243			-0.263	0.12		8	-2080.345	4176.782	0.76	0.164
**-0.152**	0.19	-0.043	-0.23	-0.083		-0.26	0.14		9	-2079.823	4177.761	1.739	0.101
**-0.154**	0.195	NA	-0.223	-0.118	0.012	-0.257	0.148		9	-2079.852	4177.818	1.796	0.098

Table S4) Comparison between full model using unstandardized variables (left) and standardized variables (right). Summary of model fit was generated by plotting the summary of the resulting phylolm object (Ho et al., 2016) on the R console. Effect sizes are in different scales, but p-values and variable importances are generally the same.

```
Call:
phylolm(formula = formula_full, data = data_subset, phy = phy,
    model = "lambda", REML = FALSE)

   AIC logLik
 779.9 -379.9

Raw residuals:
     Min       1Q   Median       3Q      Max
-1.63878 -0.14147  0.03216  0.21283  1.65020

Mean tip height: 139.3956
Parameter estimate(s) using ML:
lambda : 0.4456685
sigma2: 0.0008843848

Coefficients:
               Estimate      StdErr t.value   p.value
(Intercept)  4.4684e-01  1.2216e-01  3.6578 0.0002628 ***
bio_1       -1.5427e-02  2.6035e-03 -5.9256 3.815e-09 ***
bio_12      -3.9224e-05  3.6366e-05 -1.0786 0.2809334
bio_4       -1.9958e-04  5.2051e-05 -3.8343 0.0001309 ***
bio_15       1.2593e-04  3.7814e-04  0.3330 0.7391678
wind         1.4266e-02  1.0216e-02  1.3964 0.1627896
srad         2.0959e-05  6.1870e-06  3.3876 0.0007224 ***
ai          -3.0508e-06  4.7184e-06 -0.6466 0.5180043
---
Signif. codes:  0 ‘***’ 0.001 ‘**’ 0.01 ‘*’ 0.05 ‘.’ 0.1 ‘ ’ 1

R-squared: 0.06932      Adjusted R-squared: 0.06518

Note: p-values and R-squared are conditional on lambda=0.4456685.
```

```
Call:
phylolm(formula = formula_full, data = data_subset, phy = phy,
    model = "lambda", REML = FALSE)

   AIC logLik
  4189  -2085

Raw residuals:
    Min      1Q  Median      3Q     Max
-4.8106 -0.4153  0.0944  0.6248  4.8442

Mean tip height: 139.3956
Parameter estimate(s) using ML:
lambda : 0.4456685
sigma2: 0.007620875

Coefficients:
             Estimate    StdErr t.value   p.value
(Intercept) -0.148108  0.172790 -0.8572 0.3914898
bio_1       -0.273936  0.046229 -5.9256 3.815e-09 ***
bio_12      -0.090418  0.083830 -1.0786 0.2809334
bio_4       -0.156107  0.040713 -3.8343 0.0001309 ***
bio_15       0.008848  0.026570  0.3330 0.7391678
wind         0.044032  0.031532  1.3964 0.1627896
srad         0.136569  0.040314  3.3876 0.0007224 ***
ai          -0.052404  0.081049 -0.6466 0.5180043
---
Signif. codes:  0 ‘***’ 0.001 ‘**’ 0.01 ‘*’ 0.05 ‘.’ 0.1 ‘ ’ 1

R-squared: 0.06932      Adjusted R-squared: 0.06518

Note: p-values and R-squared are conditional on lambda=0.4456685.
```

Figure S6: Once within-species variance is considered in univariate regressions between LMA_{pred} and climatic factors, only MAT (Mean Annual Temperature) remained a significant relationship ($p<0.01$). This suggests that MAT is the only climatic variable that is robust to this added source of error, whereas other variables may be obscured by the noise introduced by within-species variation (however, it is important to note that this within-species variation should not be dismissed as merely error as it reflects genuine biological diversity).

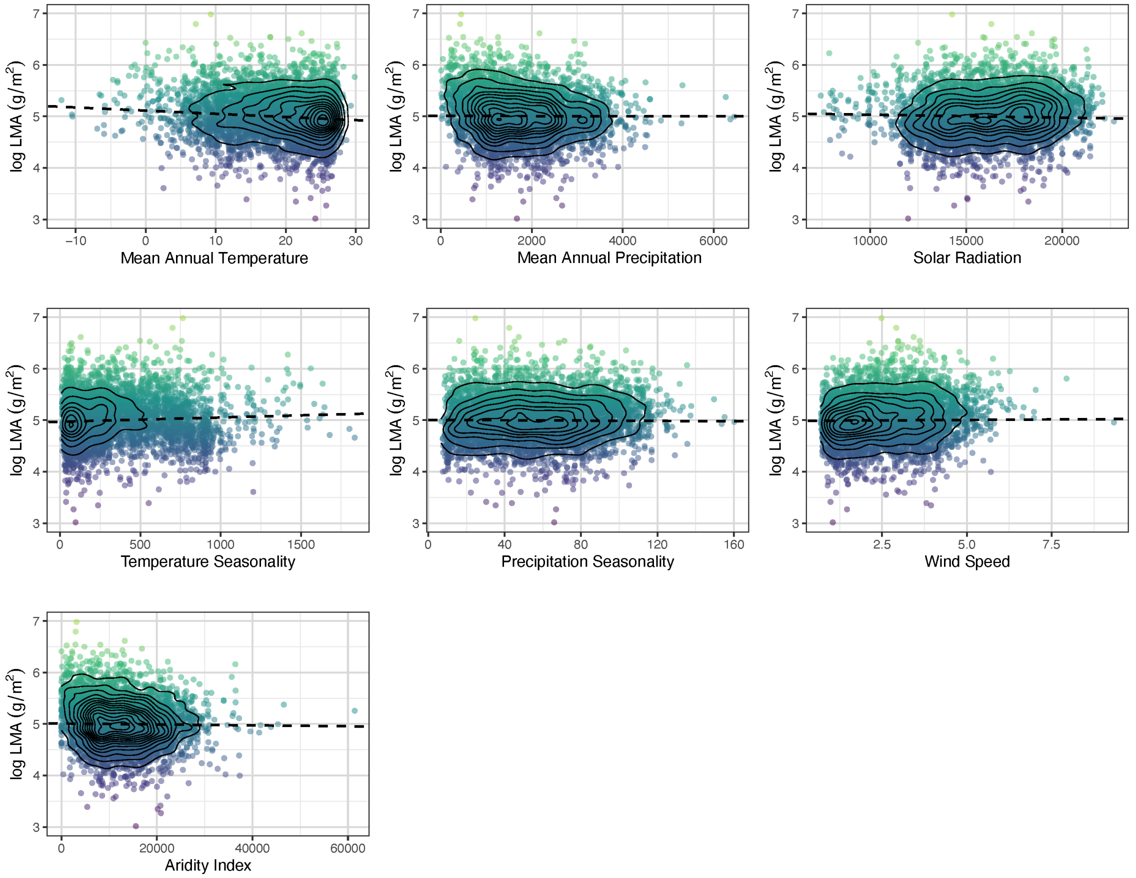

Figure S7: The map displays the geographical distribution of species in our dataset that are not currently available in the TRY database for trait 3117, "Leaf area per leaf dry mass" (Kattge et al., 2020). This comparison was conducted to evaluate the potential of our pipeline in addressing geographical and taxonomical gaps in existing open datasets of functional traits and highlight areas where our pipeline could complement existing data and enhance global trait coverage.

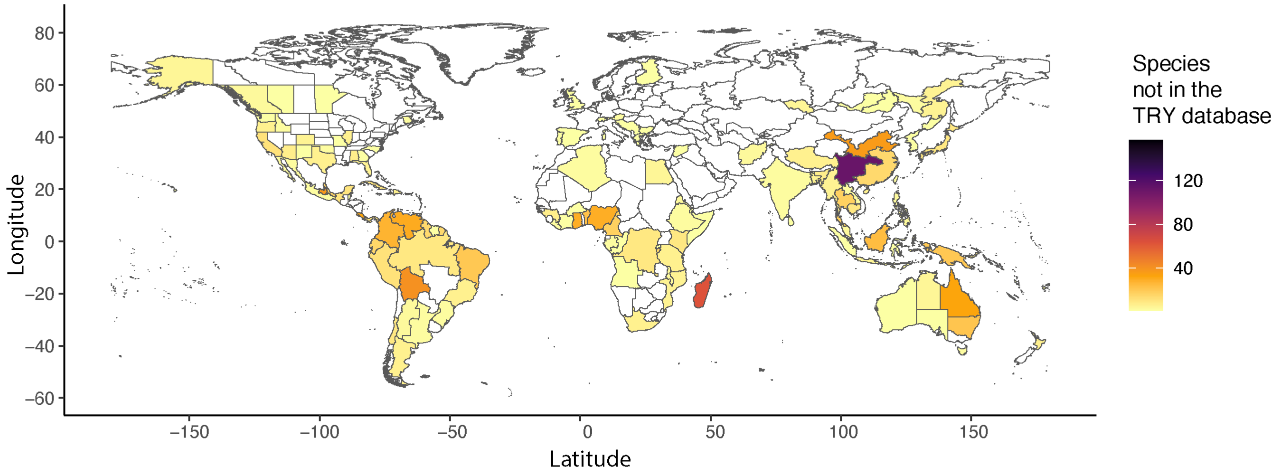

Table S5: The genera presented in this table exhibit the lowest R^2 values for the correlation between manual and LMA measurements among the genera with at least 10 successful matches between manual and automated petiole width measurements in our dataset. Upon further investigation, we found that many of these genera contain species with cordate leaves or uneven base in their leaf blades, suggesting that this leaf shape may be associated with poorer performance in our current automated models. Examples of these specimens are presented in Figure S4 below.

Genus	R^2 (manual vs. LM2 petiole width)
Berberidopsis	0.02143225
Colubrina	0.04707055
Cyathostegia	0.06414538
Crossopetalum	0.14575202
Ampelopsis	0.19746408
Betula	0.28001817
Clematis	0.28473395
Boquila	0.30997387
Alnus	0.32351312
Corylopsis	0.38774795

Figure S8: Examples of specimens in our dataset belonging to the genera identified in Table S3.

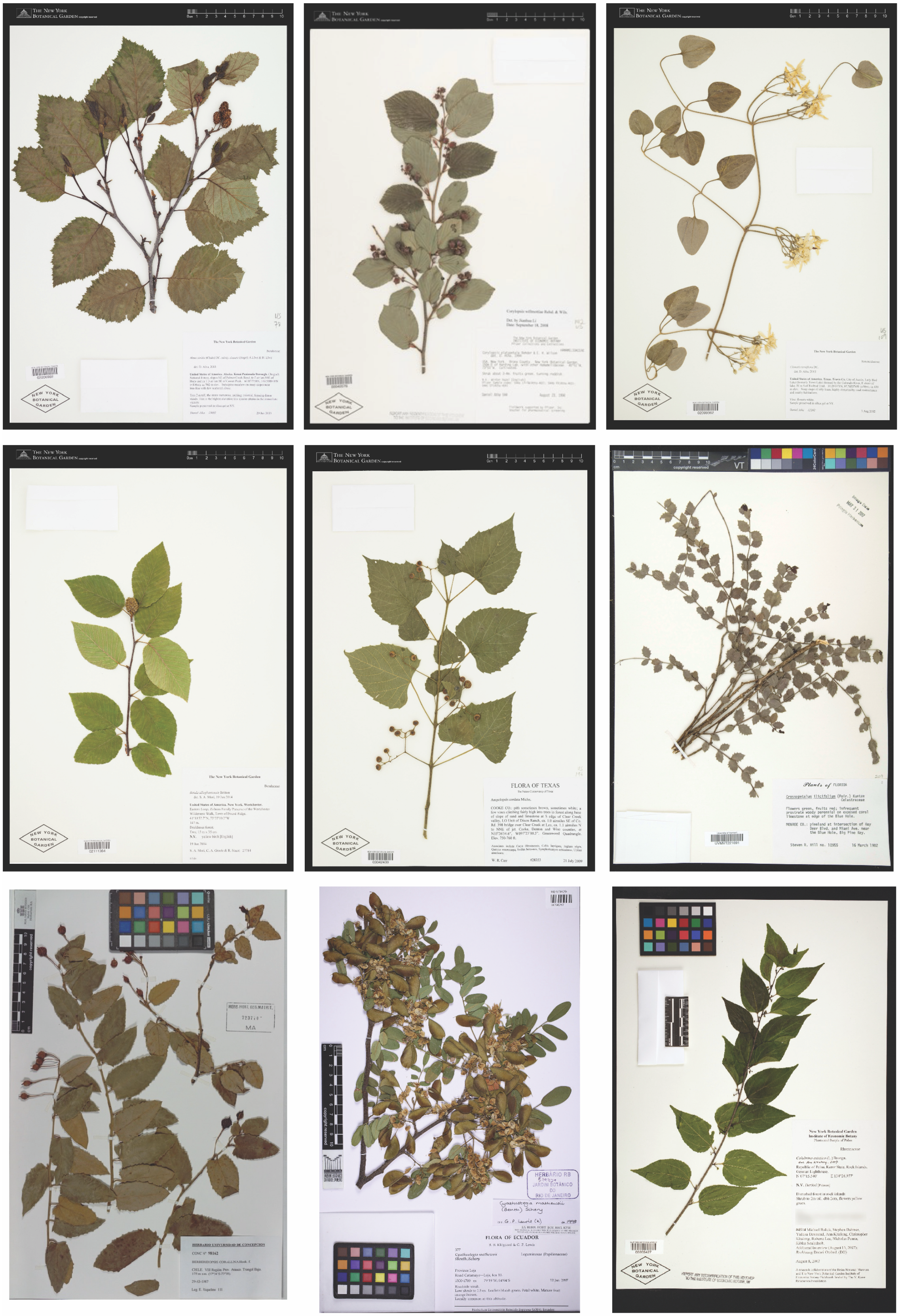

Figure S9) Because it is well known that the relationship between LMA and climate are also variable in terms of whether the species is deciduous or evergreen, we combined data available in TRY (Kattge et al., 2020), BIEN (Maitner et al., 2018) and other previously published datasets (Wright et al., 2004; Peppe et al., 2011) to gather data on leaf phenology for our species list, recovering data for 751 taxa, about half of the species. This information is available in column "leaf_phenology" of Table S2, and boxplots below show the distribution of LMA values in the two categories in log scale.

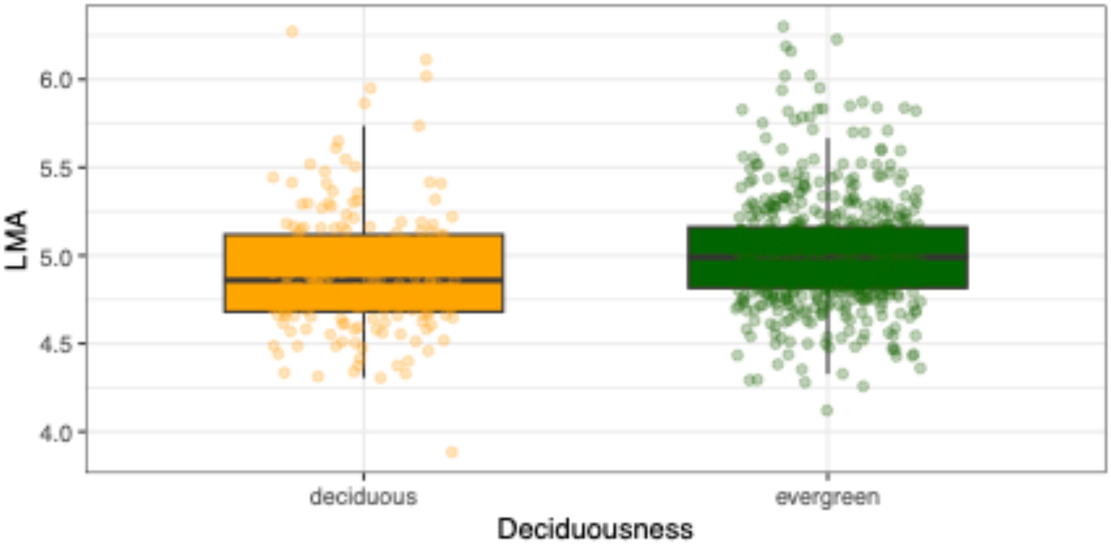

References

Ho, L.S.T., Ane, C., Lachlan, R., Tarpinian, K., Feldman, R., Yu, Q., van der Bijl, W., Maspons, J., Vos, R. and Ho, M.L.S.T., 2016. Package 'phylolm'. *See http://cran. r-project. org/web/packages/phylolm/index. html (accessed February 2018)*.

Kattge, J., Bönisch, G., Díaz, S., Lavorel, S., Prentice, I.C., Leadley, P., Tautenhahn, S., Werner, G.D., Aakala, T., Abedi, M. et al. 2020. TRY plant trait database–enhanced coverage and open access. *Global change biology*, *26*(1), pp.119-188.

Maitner, B.S., Boyle, B., Casler, N., Condit, R., Donoghue, J., Durán, S.M., Guaderrama, D., Hinchliff, C.E., Jørgensen, P.M., Kraft, N.J. et al. 2018. The bien r package: A tool to access the Botanical Information and Ecology Network (BIEN) database. *Methods in Ecology and Evolution*, *9*(2), pp.373-379.

Peppe, D.J., Royer, D.L., Cariglino, B., Oliver, S.Y., Newman, S., Leight, E., Enikolopov, G., Fernandez-Burgos, M., Herrera, F., Adams, J.M. et al. 2011. Sensitivity of leaf size and shape to climate: global patterns and paleoclimatic applications. *New Phytologist*, 190(3), pp.724-739.

Poorter H, Niinemets Ü, Poorter L, Wright IJ, Villar R. 2009. Causes and consequences of variation in leaf mass per area (LMA): a meta-analysis. *New Phytologist* 182: 565–588.

Sack L, Scoffoni C, McKown AD, Frole K, Rawls M, Havran JC, Tran H, Tran T. 2012. Developmentally based scaling of leaf venation architecture explains global ecological patterns. *Nature Communications* 3: 837.

Taylor A, Weigelt P, Denelle P, Cai L, Kreft H. 2023. The contribution of plant life and growth forms to global gradients of vascular plant diversity. *New Phytologist* 240: 1548–1560

Wright, I.J., Reich, P.B., Westoby, M., Ackerly, D.D., Baruch, Z., Bongers, F., Cavender-Bares, J., Chapin, T., Cornelissen, J.H., Diemer, M. et al. 2004. The worldwide leaf economics spectrum. *Nature*, *428*(6985), pp.821-827.

Wright IJ, Dong N, Maire V, Prentice IC, Westoby M, Díaz S, Gallagher RV, Jacobs BF, Kooyman R, Law EA et al. 2017. Global climatic drivers of leaf size. *Science* 357: pp.917–921.
